# MAP3K3 Contributes to Myocardial Ischemia/Reperfusion Injury by Promoting Myeloid Cell Diapedesis through TAL1/JAM-A Pathway

**DOI:** 10.7150/thno.122130

**Published:** 2026-01-01

**Authors:** Shiyu Hu, Jian Zhang, Jingpu Wang, Chenguang Li, Yiwen Wang, Jiayu Liang, Rong Huang, Ji'e Yang, Yang Gao, Yanan Qu, Hongbo Yang, Juying Qian, Wenwen Tang, Jiatian Cao, Feng Zhang, Junbo Ge

**Affiliations:** 1 Department of Cardiology, Shanghai Institute of Cardiovascular Diseases, Zhongshan Hospital, Fudan University, 200032 Shanghai, China.; 2 Vascular Biology and Therapeutics Program, Department of Pharmacology, Yale University School of Medicine, New Haven, CT 06520, USA.; 3 National Clinical Research Center for Interventional Medicine, 200032 Shanghai, China.; 4 State Key Laboratory of Cardiovascular Diseases, Zhongshan Hospital, Fudan University, 200032 Shanghai, China.; 5 NHC Key Laboratory of Ischemic Heart Diseases, 200032 Shanghai, China.; 6 Key Laboratory of Viral Heart Diseases, Chinese Academy of Medical Sciences, 200032 Shanghai, China.; 7 Department of Cardiology, The first Affiliated Hospital of University of Science and Technology of China (Anhui Provincial Hospital), 230000 Anhui, China.

**Keywords:** myocardial ischemia/reperfusion injury, myeloid cells, diapedesis, MAP3K3, TAL1, JAM-A

## Abstract

**Rationale:** Extensive leukocyte diapedesis is a defining step in inflammation and contributes critically to myocardial ischemia/reperfusion injury (MI/RI). Infiltrating leukocytes amplify local inflammation and exacerbate myocardial damage. However, the upstream control of the trans-endothelial migration step remains incompletely understood.

**Methods:** Peripheral blood myeloid cells were isolated from MI/RI patients and healthy donors to examine MAP3K3 expression and its correlation with cardiac markers. Mouse MI/RI models were established to investigate MAP3K3 expression of myeloid cells in the heart. Myeloid-specific *Map3k3* deficiency mice were used to evaluate the impact of MAP3K3 depletion on MI/RI severity and on myeloid cell diapedesis from the bone marrow. RNA sequencing and various manipulations of the MAP3K3/TAL1/JAM-A axis were used to elucidate its role in diapedesis. Finally, the therapeutic potential of pazopanib, a MAP3K3 inhibitor, was evaluated in the mouse MI/RI model.

**Results:** MAP3K3 expression was upregulated in both monocytes and neutrophils from MI/RI patients and was positively correlated with the severity of MI/RI. In mice, MAP3K3 in cardiac myeloid cells peaked at day 3 post-MI/RI. Myeloid cell-specific depletion of MAP3K3 alleviated MI/RI by reducing the infiltration of myeloid cells into cardiac tissue. Functionally, MAP3K3 facilitated myeloid cell de-adhesion and transmigration across endothelial barriers. Further mechanistic studies identified the MAP3K3/TAL1/JAM-A signaling pathway as a key regulator of myeloid cell diapedesis. MAP3K3 phosphorylates TAL1 at Ser-122, leading to its ubiquitination and attenuating its transcriptional repression of *F11r* (encoding JAM-A). Through JAM-A, MAP3K3 promotes integrin internalization, thereby enhancing de-adhesion and myeloid cell transmigration. Treatment with pazopanib, a MAP3K3 inhibitor, ameliorated MI/RI injury and reduced myeloid cell diapedesis into the heart by blocking MAP3K3 phosphorylation activity.

**Conclusions:** MAP3K3 orchestrates myeloid cell diapedesis via a TAL1/JAM-A dependent program during MI/RI. Targeting MAP3K3, exemplified by pazopanib, may offer a therapeutic strategy for MI/RI and related inflammatory conditions.

## Introduction

Although early restoration of blood supply reduces cardiomyocyte loss after myocardial infarction (MI) 1, secondary myocardial ischemia/reperfusion injury (MI/RI) still threatens the heart function 2. Extensive leukocyte division and subsequent leukocyte diapedesis plays a central role in different inflammatory processes, including MI/RI. While leukocyte division (e.g., hematopoietic stem cell differentiation) sustains immune cell populations, leukocyte diapedesis is a dynamic process enabling monocytes, macrophages, and neutrophils to exit vasculature toward inflammatory sites 3. Recruited leukocytes in the heart produce a large amount of pro-inflammatory factors, leading to an inflammatory cascade and myocardial injury 4.

The complete process of leukocyte diapedesis includes chemotaxis of leukocytes, leukocyte adhesion to vessels, and transmigration through vessels 5. During reperfusion, dead or injured cardiomyocytes released damage-associated molecular patterns and produced chemokines such as monocyte chemoattractant protein 1 (MCP-1) and interleukin 8, which recruited leukocytes from bones to blood and tissues together with the activation of the complement system 4. The elevation of several endothelial and cardiomyocyte-mediated adhesion factors such as P-selectin, E-selectin, intercellular adhesion molecule 1 (ICAM-1), and platelet endothelial cell adhesion molecule 1 (PECAM-1) promote the adhesion and rolling of peripheral leukocytes 6, which then infiltrate the ischemic myocardium through the vascular wall. Unveiling the key signaling pathway involved in leukocyte diapedesis offers valuable insights into the mechanisms underlying severe inflammation during MI/RI and presents potential therapeutic targets for its treatment.

Mitogen-activated protein kinase kinase kinase 3 (MAP3K3) is a highly conserved member of the MAP3K superfamily with Ser/Thr protein kinase activity 7. MAP3K3 is involved in multiple important biological pathways: activation of ERK5 and p38 in the MAPK pathway 7-9, regulation of toll-like receptor pathway and NF-κB pathway 10, 11, lymphocyte differentiation 12, and development of the cardiovascular system and cerebrovascular disease 13-15. MAP3K3 is reported as a key kinase in different cell types and inflammatory processes, such as neutrophils in acute lung injury 16, and platelets in MI 17, 18. MAP3K3 in neutrophil mainly influences reactive oxygen species (ROS) formation 16, while MAP3K3 in platelets participates in thrombosis 17. However, the role of MAP3K3 in myeloid cells, especially the biological process of diapedesis, is still unknown.

T cell acute lymphocytic leukemia (TAL1) is reported to play a vital role in the generation of erythroid and myeloid lineages 19, and its overexpression in lymphocytes can cause acute lymphoblastic leukemia 20. Its function in the diapedesis of myeloid cells remains unclear.

Junctional adhesion molecule-A (JAM-A, encoded by *F11r* gene) is primally located in the tight junction between endothelial cells 21. JAM-A on platelets has also been found to be involved in platelet-leukocyte aggregation 22, 23. A few studies have found its role in leukocytes as a regulator of cell polarization 24 and transmigration through blood vessels 25. JAM-A controls integrin internalization, promotes the de-adhesion of leukocytes from endothelial cells, and leads to the transmigration of leukocytes through blood vessels 25. Deficiency of JAM-A can reduce leukocyte diapedesis during hepatic ischemia/reperfusion injury 26, MI/RI 27, acute lung injury 28, and atherosclerosis 29.

In this study, we disclosed the function of MAP3K3 on leukocyte diapedesis and unveiled the regulation function of MAP3K3 on JAM-A through the phosphorylation and ubiquitination of TAL1 on serine 122. Targeting MAP3K3 or JAM-A can both influence leukocyte diapedesis. Deficiency of MAP3K3 in myeloid cells or administration of pazopanib (an inhibitor of MAP3K3 16) attenuated MI/RI by decreasing leukocyte diapedesis. We provided a new insight into the MAP3K3/TAL1/JAM-A pathway in leukocyte diapedesis, highlighting its potential as a therapeutic target not only for MI/RI but also for other inflammatory diseases.

## Methods

All data, study methods and materials that support the findings of this study are available from the corresponding authors on reasonable request. Detailed methods are provided in the Supplementary Material.

### Patients

Peripheral blood of the MI/RI group was obtained 12 hours after PCI in 66 patients diagnosed with acute coronary syndrome (ACS) (clear diagnosis of myocardial infarction, STEMI and non-STEMI, and the presence of culprit vessels, stenosis greater than 90%, on coronary angiography). Peripheral blood of the control group was obtained from 6 donors with negative coronary angiography results (have coronary artery stenosis less than 50% and no other diseases that clearly affected coronary artery function) 30. The study was approved by the Ethics Committee of the Fudan University Zhongshan Hospital, China (approval number: B2021-754). Informed consent was received from all patients as per the Declaration of Helsinki.

### Animal study design and establishment of MI/RI models

Mouse MI/RI model was constructed with a 45-minute ligation time at the left anterior descending coronary artery 31.

The *Map3k3*^fl/fl^ and *Map3k3*^CKO^ mice were constructed by Cyagen Biosciences in C57BL/6 mice (*Lyz2*-Cre mice were from Jackson Laboratory Bar, Harbor, ME). Littermate mice with average age of 6-8 weeks were used for all experiments.

Sex-matched wild-type (WT) mice (Shanghai Jiesijie) aged 6-8 weeks were randomly divided into the saline group (200 μL saline) and pazopanib-treated group. Pazopanib-treated mice were injected intraperitoneally with 1.5 mg/kg pazopanib per mouse as previously described 16, 32. The mice were injected immediately after surgery (after ligation, before reperfusion), 24 hours after surgery, and 48 hours after surgery.

All animal experiments were conducted following the ARRIVE guidelines and approved by the Animal Care and Use Committee (approval number: 2023-196, Zhongshan Hospital, Fudan University).

### Statistical analysis

Statistical analyses were performed using GraphPad Prism software (version 8.4.2), following previously described methods 33. For datasets involving two or more independent factors, comparisons were made using two-way ANOVA, while categorical variables were analyzed by chi-square test. A *P* value of less than 0.05 was considered statistically significant.

## Results

### MAP3K3 expression in myeloid cells increased after MI/RI

**Figure 1:**
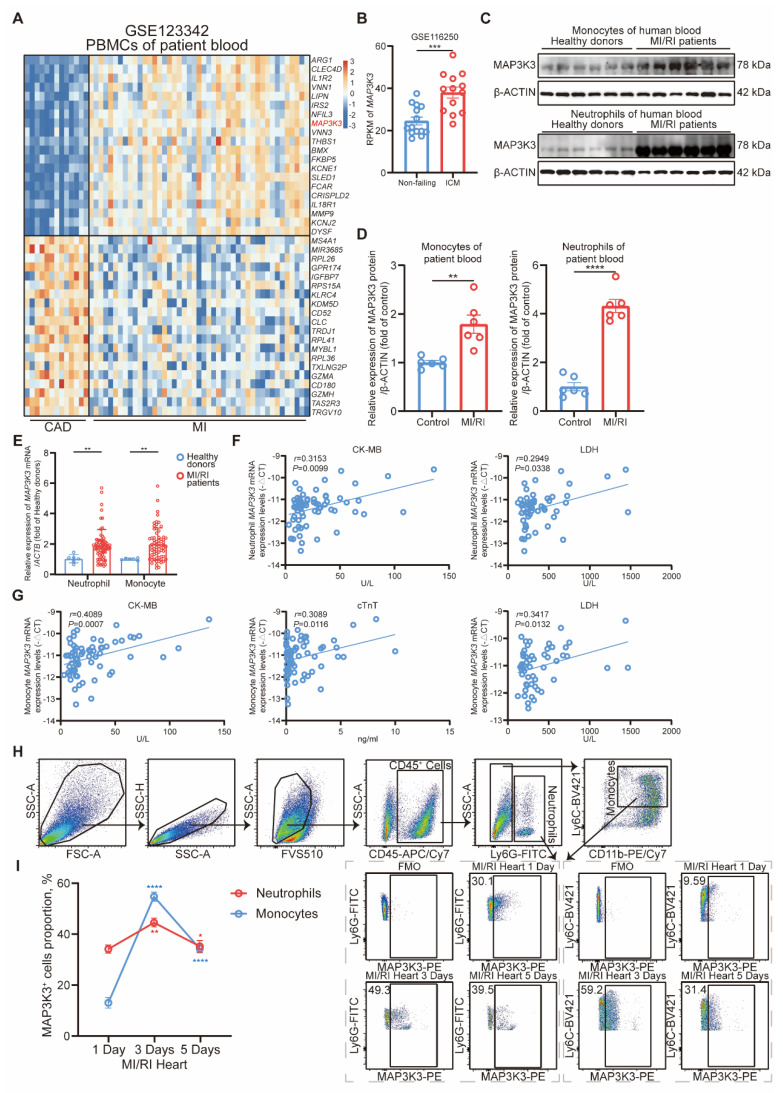
** MAP3K3 expression in myeloid cells increased and correlated with cardiac markers during MI/RI. (A)** Heatmap of the differential expression genes (DEGs) in peripheral blood mononuclear cells (PBMCs) from myocardial infarction (MI) patients (*n*=45) compared to stable coronary artery disease (CAD) patients (*n*=14) (GSE123342). **(B)** *MAP3K3* gene expression levels in human left ventricle tissue from ischemic cardiomyopathy (*n*=13) compared to non-failing donors (*n*=14) (GSE116250, unpaired *t* test). **(C-D)** Relative protein levels of MAP3K3 of monocytes (unpaired *t* test with Welch's correction) and neutrophils (unpaired *t* test) from myocardial ischemia/reperfusion injury (MI/RI) patients and healthy donors, respectively (n=6 each). **(E)** Relative mRNA levels of *MAP3K3* of neutrophils and monocytes from MI/RI patients (*n*=66 each) and healthy donors (*n*=6 each), respectively (Mann-Whitney test for both). **(F)** Spearman correlation analysis of *MAP3K3* mRNA levels in neutrophils with creatine kinase MB isoenzyme (CK-MB, *n*=66) and lactate dehydrogenase (LDH, *n*=52) levels in plasma of patients with MI/RI. **(G)** Pearson correlation analysis of *MAP3K3* mRNA levels in monocytes with CK-MB (*n*=66), cardiac troponin T (cTnT, *n*=66), and LDH (*n*=52) levels in plasma of patients with MI/RI. **(H)** Flowcytometric dot plot analysis and staining strategy for neutrophils (CD45^+^Ly6G^+^) and monocytes (CD45^+^Ly6G^-^CD11b^+^Ly6C^+^) in MI/RI tissues and MAP3K3 expression levels in either myeloid cell. **(I)** MAP3K3 expression levels of neutrophils or monocytes after MI/RI in different time (*n*=4, one-way ANOVA test with Tukey's multiple comparisons test, each). All data were displayed as mean ± SEM. * *P* < 0.05; ** *P* < 0.01; *** *P* < 0.001; **** *P* < 0.0001.

Through a bioinformatic transcriptome analysis on GSE123342 34, we found *MAP3K3* expression levels increased significantly in peripheral blood mononuclear cells (PBMCs) from MI patients compared to stable coronary artery disease (CAD) patients (Figure 1A). In human left ventricle tissue ischemic cardiomyopathy (ICM, GSE57338 35), *MAP3K3* was also upregulated (Figure 1B). We validated this upregulation of MAP3K3 in neutrophils and monocytes from MI/RI patients respectively (Figure 1C-E). The age, sex, and morbidity of hypertension, diabetes, hyperlipidemia, and stroke between MI/RI patients and the Control group differed insignificantly, suggesting that the elevated expression level of MAP3K3 was mainly caused by MI/RI (detailed in Table 1).

To further explore the relationship between MAP3K3 and the severity of MI/RI, we assessed the relationship between *MAP3K3* expression level in neutrophils and monocytes of MI/RI patients and serum cardiac markers 36, 37, including CK-MB (creatine kinase M-type), cTnT (cardiac troponin T), LDH (lactate dehydrogenase), pro-BNP (pro B-type natriuretic peptide), CRP (C-reactive protein), IL-6 (interleukin-6), and complete blood count. We found that mRNA expression levels of *MAP3K3* in neutrophils and monocytes had a positive correlation with CK-MB, and LDH, while *MAP3K3* in monocytes also had a positive correlation with cTnT (Figure 1F-G). The mRNA expression levels of *MAP3K3* in neutrophils and monocytes also had a positive correlation with mRNA expression levels of *IL-6*, indicating that MAP3K3 might aggravate inflammation in MI/RI patients (Figure S1A-B).

To explore the expression level of MAP3K3 in infiltrating myeloid cells in cardiac tissue after MI/RI, we conducted flowcytometric dot plot analysis on cardiac tissue after MI/RI at different time points (Figure 1H and Figure S1C). We found that MAP3K3 expression levels peaked at 3 days after MI/RI in myeloid cells (Figure 1I). Immunofluorescence staining of MI/RI tissue sections also showed large amounts of infiltrating myeloid cells in heart tissue with high expression levels of MAP3K3 (Figure S1D).

### Myeloid-specific *Map3k3* deficiency alleviates MI/RI through dysfunction of diapedesis from bone marrow

**Figure 2:**
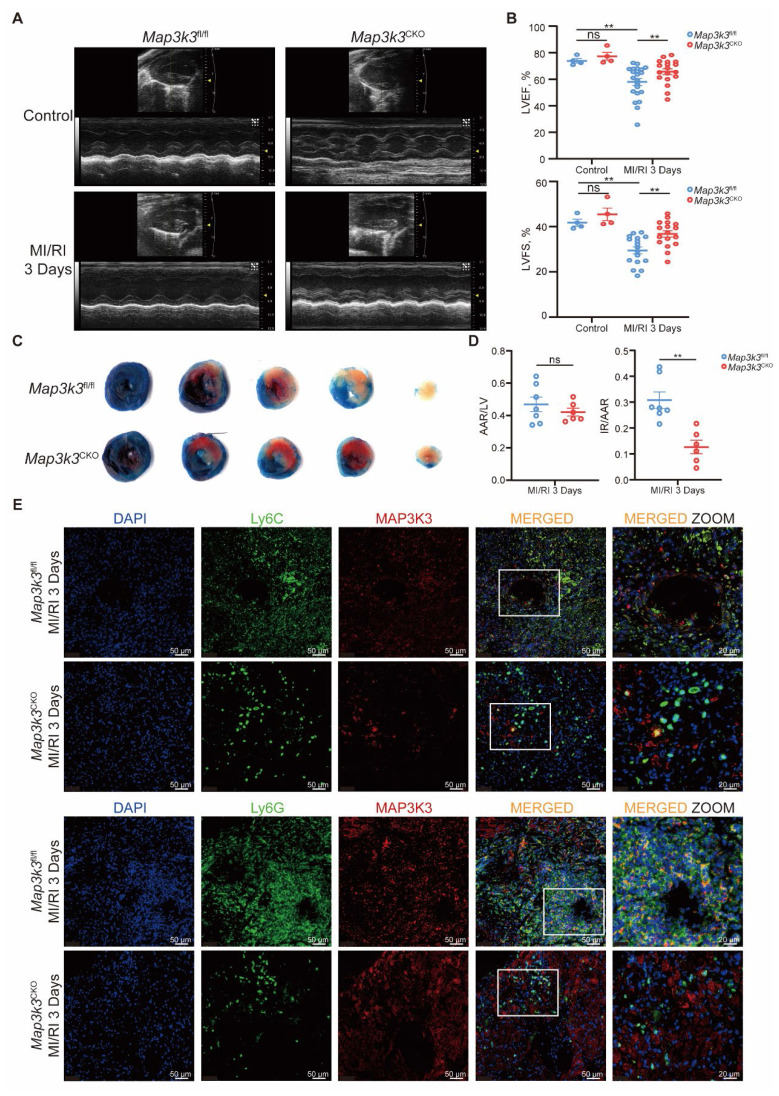
** Myeloid-specific *Map3k3* deficiency alleviated MI/RI and decreased myeloid cell infiltration to heart. (A)** Representative images of echocardiography of *Map3k3*^CKO^ mice and *Map3k3*^fl/fl^ mice undergoing MI/RI. **(B)** Left ventricular ejection fraction (LVEF, top) and left ventricular fractional shortening (LVFS, bottom, one-way ANOVA test with Tukey's multiple comparisons test for both) of control + *Map3k3*^fl/fl^ mice (*n*=4), control + *Map3k3*^CKO^ mice (*n*=4), MI/RI + *Map3k3*^fl/fl^ mice (*n*=17), and MI/RI + *Map3k3*^CKO^ mice (*n*=17). **(C)** Representative image of left ventricular tissue sections stained with Evans blue and 2,3,5-triphenyl tetrazolium chloride at 3 days after MI/RI to delineate the area at risk (AAR, red) and the infarcted area (IR, white). **(D)** The ratios of AAR/LV (left) and IR/AAR (right, unpaired *t* test for both) were compared (*n*=7 for *Map3k3*^fl/fl^ mice, *n*=6 for *Map3k3*^CKO^ mice). **(E)** Representative fluorescence images of myeloid cells infiltrated in MI/RI tissue sections stained for DAPI (blue), Ly6G/Ly6C (green), MAP3K3 (red), *200, scale bar 50 μm; *400, scale bar 20 μm. All data were displayed as mean ± SEM. ns, *P* > 0.05; ** *P* < 0.01.

We next generated myeloid-specific *Map3k3* deficiency (*Map3k3*^CKO^) mice and conducted MI/RI models in *Map3k3*^CKO^ mice compared to *Map3k3*^fl/fl^ mice. Deficiency of *Map3k3* in myeloid cells preserved heart function reflected by elevated left ventricular ejection fraction (LVEF) and left ventricular fraction shortening (LVFS) (Figure 2A-B) and reduced infarcted size (Figure 2C-D). Expression levels of apoptotic protein BAX and cleaved CASPASE-3 also decreased in *Map3k3*^CKO^ mice (Figure S2A-B). Deficiency of *Map3k3* in myeloid cells also reduced apoptosis of cardiomyocytes stained by TdT-mediated dUTP nick end labeling (TUNEL, Figure S2C).

**Figure 3:**
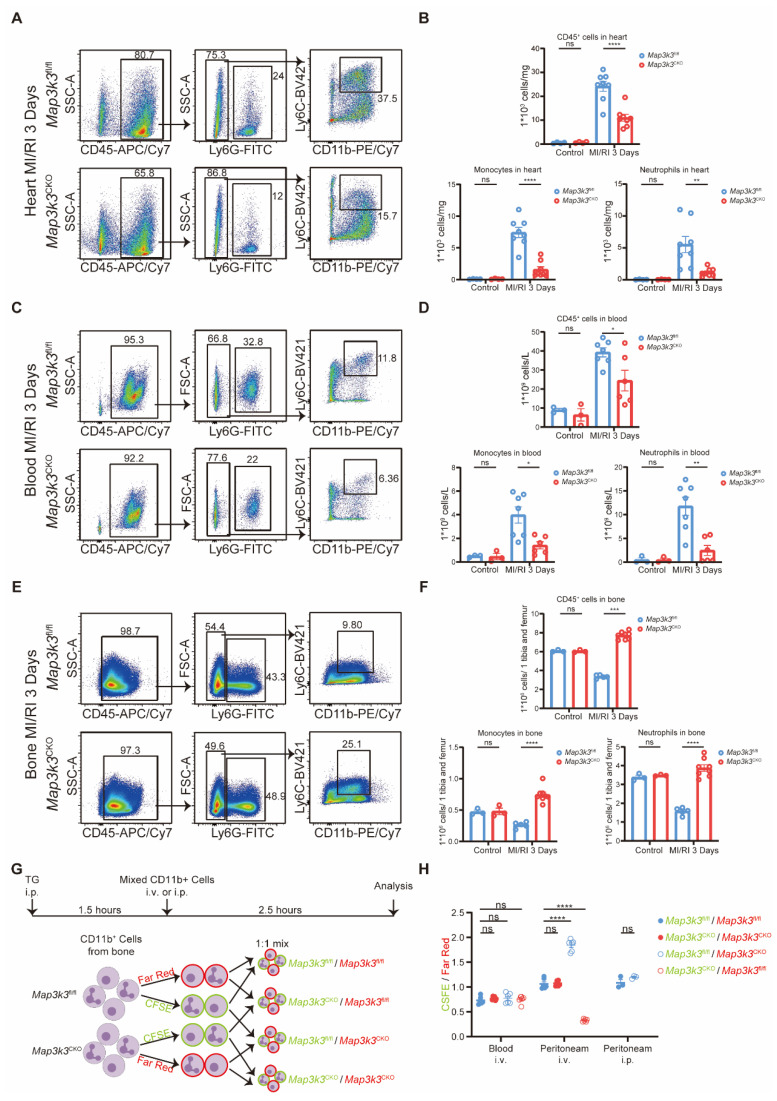
** Myeloid-specific *Map3k3* deficiency decreased diapedesis from bone after MI/RI and diapedesis *in vivo*. (A)** Representative flowcytometric dot plot analysis of infiltrating myeloid cells in heart of *Map3k3*^fl/fl^ mice and* Map3k3*^CKO^ mice. **(B)** Proportion of leukocytes (top), monocytes (bottom left), and neutrophils (bottom right, one-way ANOVA test with Tukey's multiple comparisons test for all) in heart of control + *Map3k3*^fl/fl^ mice (*n*=4), control + *Map3k3*^CKO^ mice (*n*=4), MI/RI + *Map3k3*^fl/fl^ mice (*n*=8), and MI/RI + *Map3k3*^CKO^ mice (*n*=8). **(C)** Representative flowcytometric dot plot analysis of myeloid cells in blood of *Map3k3*^fl/fl^ mice and* Map3k3*^CKO^ mice. **(D)** Proportion of leukocytes (top, one-way ANOVA test with Tukey's multiple comparisons test), monocytes (bottom left, Kruskal-Wallis's test with Dunn's multiple comparisons test), and neutrophils (bottom right, one-way ANOVA test with Tukey's multiple comparisons test) in blood of control + *Map3k3*^fl/fl^ mice (*n*=4), control + *Map3k3*^CKO^ mice (*n*=4), MI/RI + *Map3k3*^fl/fl^ mice (*n*=7), and MI/RI + *Map3k3*^CKO^ mice (*n*=6).** (E)** Representative flowcytometric dot plot analysis of myeloid cells in bone of *Map3k3*^fl/fl^ mice and* Map3k3*^CKO^ mice. **(F)** Proportion of leukocytes (top, Kruskal-Wallis's test with Dunn's multiple comparisons test), monocytes (bottom left, one-way ANOVA test with Tukey's multiple comparisons test), and neutrophils (bottom right, one-way ANOVA test with Tukey's multiple comparisons test) in bone of control + *Map3k3*^fl/fl^ mice (*n*=4), control + *Map3k3*^CKO^ mice (*n*=4), MI/RI + *Map3k3*^fl/fl^ mice (*n*=5), and MI/RI + *Map3k3*^CKO^ mice (*n*=8). **(G)** Study design of myeloid cells diapedesis *in vivo* experiment. Mice were intraperitoneally injected (i.p.) with thioglycolate (TG) for 1.5 hours. Bone marrow myeloid cells were labeled with a CFSE or a far-red dye and tail vein injected (i.v.) or intraperitoneally injected (i.p.) to mice with peritonitis. Cells were collected 2.5 hours afterward from peripheral blood and peritoneum. **(H)** The ratios between CFSE and far-red-labeled cells collected from blood after i.v. (*n*=6 each, Kruskal-Wallis's test with Dunn's multiple comparisons test), from peritoneum after i.v. (*n*=6 each, one-way ANOVA test with Tukey's multiple comparisons test), and from peritoneum after i.p. (*n*=3 each, unpaired *t* test). All data were displayed as mean ± SEM. ns, *P* > 0.05; * *P* < 0.05; ** *P* < 0.01; *** *P* < 0.001; **** *P* < 0.0001.

To explore the function of MAP3K3 in myeloid cells during MI/RI, we first conducted immunofluorescence staining of MI/RI tissue sections and found that deficiency of *Map3k3* in myeloid cells led to a decrease of infiltrating myeloid cells in heart tissue (Figure 2E and Figure S2D). To further elucidate this diapedesis change caused by deficiency of *Map3k3* in myeloid cells, we conducted flowcytometric dot plot analysis on MI/RI mice (Figure S3A). We found that *Map3k3*^CKO^ mice showed a decreased proportion of both neutrophils and monocytes infiltrated in the heart (Figure 3A-B and Figure S1C). Tracing back along the diapedesis of myeloid cells, we found that neutrophils and monocytes also decreased in the blood of *Map3k3*^CKO^ mice (Figure 3C-D and Figure S3B) but increased in the bone of *Map3k3*^CKO^ mice (Figure 3E-F and Figure S3B). Given that myeloid cells didn't home to the spleen (Figure S3C-D), we concluded that MAP3K3 might regulate the diapedesis of myeloid cells from bone to blood and eventually to heart. Hematoxylin-eosin (HE) staining also showed less diapedesis and disorganization in the heart of *Map3k3*^CKO^ mice (Figure S3E).

In addition, we conducted another inflammatory model, lipopolysaccharide (LPS)-induced sepsis, and examined diapedesis in targeted tissue lung and heart. As LPS-induced sepsis is an acute inflammation model, we focused on the diapedesis of neutrophils. As expected, deficiency of *Map3k3* in myeloid cells decreased the diapedesis of neutrophils to the lung and heart from blood and bone (Figure S4).

We also performed another *in vivo* experiment as previously reported 38 to determine the function of MAP3K3 in diapedesis. After pre-treating mice with thioglycolate (TG) for 1.5 hours, a mixture of labeled myeloid cells from the bone of *Map3k3*^CKO^ mice or *Map3k3*^fl/fl^ mice was injected into circulation, with the ratio between the two myeloid cells populations assessed in the peritoneum and blood after 2.5 h (Figure 3G). All mixtures showed a mildly different label ratio in the blood, which confirmed the 1:1 mixture and no influence during the injection process. In the peritoneum, we observed a reduced proportion of myeloid cells of *Map3k3*^CKO^ mice regardless of dye. While myeloid cells were injected directly into the peritoneum, we didn't observe significant changes in the proportion of myeloid cells (Figure 3H).

To this extent, we found that MAP3K3 played a vital role in the diapedesis of myeloid cells.

### Myeloid-specific *Map3k3* deficiency increased inhibitory transcription function of TAL1 on *F11r* by phosphorylation

**Figure 4:**
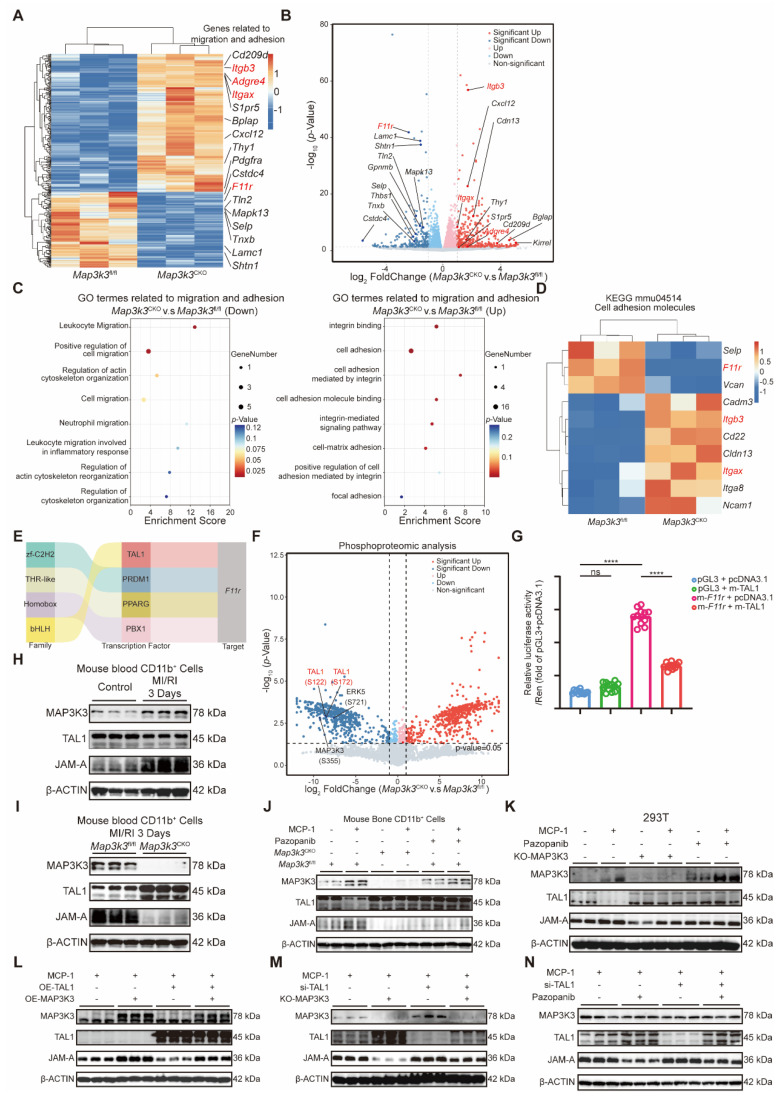
** Myeloid-Specific *Map3k3* Deficiency increased inhibitory transcription function of TAL1 on *F11r*. (A)** Heatmap of the DEGs in bone marrow myeloid cells from *Map3k3*^fl/fl^ mice (*n*=3) and *Map3k3*^CKO^ mice (*n*=3). Genes related to migration and adhesion were shown. **(B)** Volcano plot of the DEGs in bone marrow myeloid cells from *Map3k3*^fl/fl^ mice (*n*=3) and *Map3k3*^CKO^ mice (*n*=3). Genes related to migration and adhesion were shown. **(C)** GO analysis of DEGs. GO terms related to migration and adhesion were shown. **(D)** Heatmap of the DEGs from KEGG term 'Cell adhesion molecules' (mmu04514). **(E)** Four candidate transcription factors potentially involved in the regulation of *F11r* transcription predicted by RNA-seq. **(F)** Volcano plot of differentially expressed phosphorylated protein of phosphoproteomic analysis comparing bone marrow myeloid cells from *Map3k3*^fl/fl^ mice (*n*=3) and *Map3k3*^CKO^ mice (*n*=3). Phosphorylated protein related to MAP3K3 and candidate transcription factors were shown. **(G)** TAL1 and *F11r* promoter luciferase activity were detected by dual-luciferase assays (*n*=12 each, Brown-Forsythe test with Dunnett's T3 multiple comparisons test). **(H)** Western blots of MAP3K3, TAL1, and JAM-A proteins from blood myeloid cells of control mice and MI/RI mice. **(I)** Western blots of MAP3K3, TAL1, and JAM-A proteins from blood myeloid cells of *Map3k3*^fl/fl^ mice and *Map3k3*^CKO^ mice after MI/RI. **(J)** Western blots of MAP3K3, TAL1, and JAM-A proteins from bone marrow myeloid cells of *Map3k3*^fl/fl^ mice and *Map3k3*^CKO^ mice, treated with MCP-1 and Pazopanib or not. **(K)** Western blots of MAP3K3, TAL1, and JAM-A proteins from 293T, treated with MCP-1 and Pazopanib or not, transfected with MAP3K3 knockdown adenovirus or not. **(L)** Western blots of MAP3K3, TAL1, and JAM-A proteins from 293T, treated with MCP-1, transfected with TAL1 overexpression plasmid and MAP3K3 overexpression plasmid or not. **(M)** Western blots of MAP3K3, TAL1, and JAM-A proteins from 293T, treated with MCP-1, transfected with TAL1 siRNA and MAP3K3 knockdown adenovirus or not. **(N)** Western blots of MAP3K3, TAL1, and JAM-A proteins from 293T, treated with MCP-1, transfected with TAL1 siRNA, and Pazopanib or not. All data were displayed as mean ± SEM. ns, *P* > 0.05; **** *P* < 0.0001.

To elucidate the downstream mechanism of MAP3K3 on diapedesis, we conducted RNA-seq comparing bone marrow myeloid cells from *Map3k3*^CKO^ mice and *Map3k3*^fl/fl^ mice. Sequencing results showed that many adhesion-related genes, such as *Itgb3*, *Adgre4*, and *Itgax*, were upregulated in *Map3k3*^CKO^ mice, while the de-adhesion gene *F11r* was downregulated in *Map3k3*^CKO^ mice (Figure 4A-B). Gene ontology (GO) analysis of the adhesion and migration pathway also showed increased enrichment of genes in adhesion-related terms and decreased enrichment of genes in migration-related genes from *Map3k3*^CKO^ mice (Figure 4C). Genes of Kyoto Encyclopedia of Genes and Genomes (KEGG) term 'Cell adhesion molecules' (mmu04514) also showed upregulation of *Itgb3* and *Itgax* and downregulation of *F11r* (Figure 4D). According to the function of JAM-A, we hypothesized that MAP3K3 regulated *F11r* expression, facilitated de-adhesion of myeloid cells to endothelial cells, and caused diapedesis.

As the change of *F11r* was at the RNA levels, we traced back to its transcription factors and identified four candidate transcription factors potentially involved in the regulation of *F11r* transcription: TAL1, PRDM1, PPARG, and PBX1 (Figure 4E). To further elucidate the functional impact and downstream effects of MAP3K3, we performed phosphoproteomic analysis comparing bone marrow myeloid cells from *Map3k3*^CKO^ mice and *Map3k3*^fl/fl^ mice. The reduced phosphorylation at MAP3K3-S355 and its known downstream ERK5-S721 39 in *Map3k3*^CKO^ cells confirmed that MAP3K3 deletion impairs its phosphorylation-mediated signaling. Among the four transcription factors, only TAL1 showed reduced phosphorylation, specifically at S122 and S172, in *Map3k3*^CKO^ cells (Figure 4F). Based on these findings, we hypothesized that MAP3K3 may regulate the transcription of *F11r* by modulating the phosphorylation of TAL1. We conducted dual-luciferase assays and found that TAL1 mainly suppressed the transcription of *F11r* (Figure 4G), as previously reported for the transcription inhibition function of TAL1 40. In myeloid cells from the blood of MI/RI mice (Figure S5A), we observed increasing expression levels of MAP3K3 and JAM-A, while TAL1 was inhibited (Figure 4H and Figure S5B). In *Map3k3*^CKO^ mice, the above expression status was reversed (Figure 4I and Figure S5C). *In vitro*, bone marrow myeloid cells stimulated by monocyte chemokine MCP-1 can also cause an increase of MAP3K3 and JAM-A with a decrease of TAL1. Cells from *Map3k3*^CKO^ mice or treated with pazopanib (a kinase inhibitor of MAP3K3 16) showed higher TAL1 expression and suppressed JAM-A (Figure 4J). To simplify the following experiments, the 293T cell line was used and we observed the same phenomenon. In 293T cells (Figure 4K), overexpressed TAL1 could reverse the up-regulation of JAM-A by overexpression of MAP3K3 (Figure 4L and Figure S5D), and the knockdown of TAL1 could reverse the down-regulation of JAM-A by MAP3K3 knockout or administration of pazopanib (Figure 4M-N and Figure S5E-F). The reverse experiments confirmed that MAP3K3 up-regulated *F11r* expression by downregulating its inhibitory transcription factor TAL1. This mechanism might be dependent on the kinase activity of MAP3K3 because the pazopanib showed a similar effect as that of MAP3K3 knockout.

### *Map3k3* knockout increased adhesion and decreased diapedesis by decreasing JAM-A expression and integrin internalization

**Figure 5:**
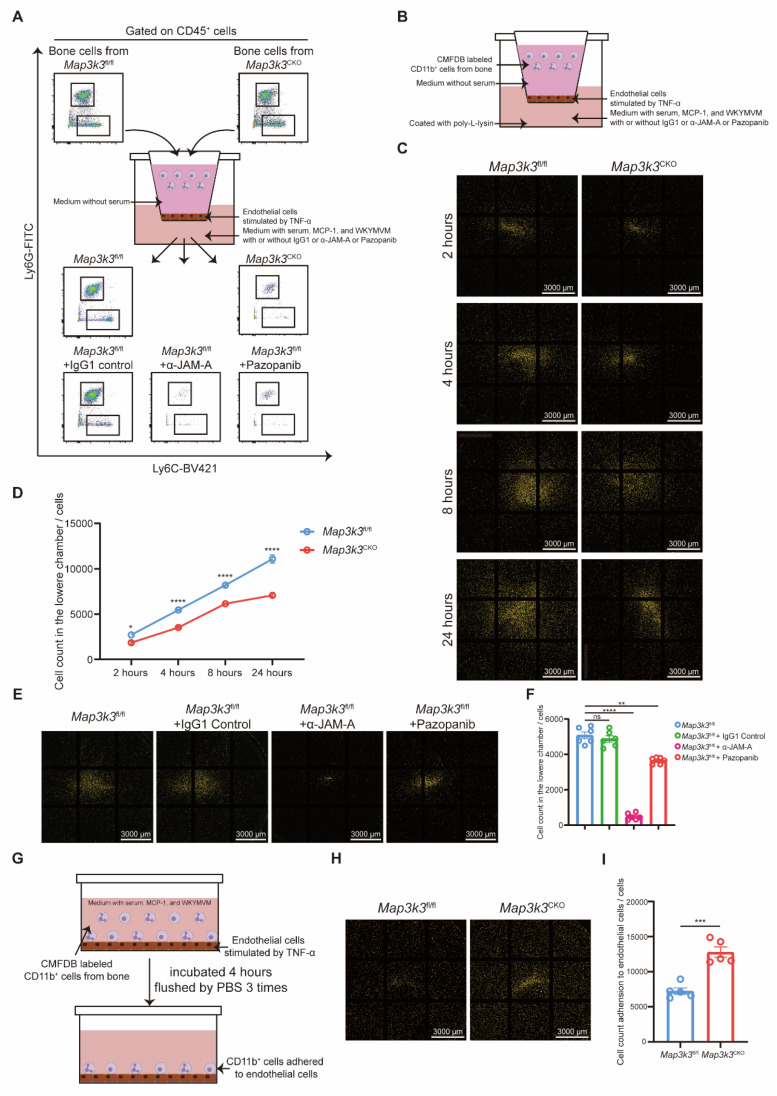
***Map3k3* Deficiency increased adhesion and decreased diapedesis through JAM-A in a phosphor-related manner. (A)** Study design of Transwell assay for flowcytometric dot plot analysis on upper chamber and lower chamber myeloid cells. **(B)** Study design of Transwell assay for microscope photography. **(C)** Representative image of lower chamber cells from *Map3k3*^fl/fl^ mice and *Map3k3*^CKO^ mice after different time course during Transwell assay. Scale bar: 3000 μm. **(D)** Cell counts in the lower chamber after different time course during Transwell assay (*n*=6 each, Two-way ANOVA test). **(E)** Representative image of lower chamber cells from *Map3k3*^fl/fl^ mice treated with IgG1, α-JAM-A, or Pazopanib after Transwell assay. Scale bar: 3000 μm. **(F)** Cell counts in the lower chamber after Transwell assay (*n*=6 each, Brown-Forsythe test with Dunnett's T3 multiple comparisons test). **(G)** Study design of Adhesion assay for microscope photography. **(H)** Representative image of adhered cells from *Map3k3*^fl/fl^ mice and *Map3k3*^CKO^ mice after Adhesion assay. Scale bar: 3000 μm. **(I)** Cell count adhesion to endothelial cells after Adhesion assay (*n*=6 each, unpaired *t* test). All data were displayed as mean ± SEM. ns, *P* > 0.05; * *P* < 0.05; ** *P* < 0.01; *** *P* < 0.001; **** *P* < 0.0001.

Next, we focused on the function of MAP3K3 in the de-adhesion of myeloid cells in the manner of phosphorylation and regulation of JAM-A *in vitro*. We conducted a Transwell assay to evaluate the transmigration of myeloid cells through endothelial cells with different treatments. Compared to the myeloid cells from *Map3k3*^fl/fl^ mice, myeloid cells from *Map3k3*^CKO^ mice showed an impaired function of diapedesis during the time course (Figure 5A-D). Meanwhile, α-JAM-A antibody or pazopanib-treated cells also showed decreased diapedesis compared to the untreated or IgG1-treated cells (Figure 5A, E-F). However, in the adhesion assay, myeloid cells from *Map3k3*^CKO^ mice showed an increased adhesion (Figure 5G-I).

As JAM-A promotes diapedesis by controlling integrin internalization 25, we tested this function *in vitro*. Flowcytometric dot plot analysis without fixation and permeabilization mainly stained the surface protein, and we found that *Map3k3* knockout, α-JAM-A, or pazopanib treatment showed higher integrin levels on plasma membrane after stimulated by chemokines, especially in neutrophils, but JAM-A on plasma membrane didn't change significantly (Figure S6A-D). Flowcytometric dot plot analysis after fixation and permeabilization showed the overall expression levels of protein in the cells. We found that *Map3k3* knockout or pazopanib treatment showed higher integrin expression levels and lower JAM-A expression levels after being stimulated by chemokines, however, α-JAM-A treatment didn't influence the expression level of integrin and JAM-A significantly (Figure S6E-H). Immunofluorescence staining of chemokines stimulated cells showed that JAM-A was able to internalize integrins in normal conditions, while *Map3k3* knockout or pazopanib treatment decreased JAM-A expression and kept integrin on the plasma membrane. α-JAM-A treatment kept JAM-A and integrin together on the plasma membrane as a complex without influencing the expression of JAM-A (Figure S6I). Plasma membrane-cytosol separation western blots also showed integrin internalization after MCP-1 stimulation which can be decreased by *Map3k3* knockout or pazopanib treatment (Figure S6J).

Immunofluorescence staining of MI/RI tissue sections showed that after depletion of *Map3k3*, myeloid cells tended to adhere to the blood vessels instead of migrating through blood vessels, resulting in the decrease of infiltrating myeloid cells in heart tissue (Figure S7A).

### MAP3K3 induced the proteasome-dependent degradation of TAL1 by phosphorylation on Ser-122

**Figure 6:**
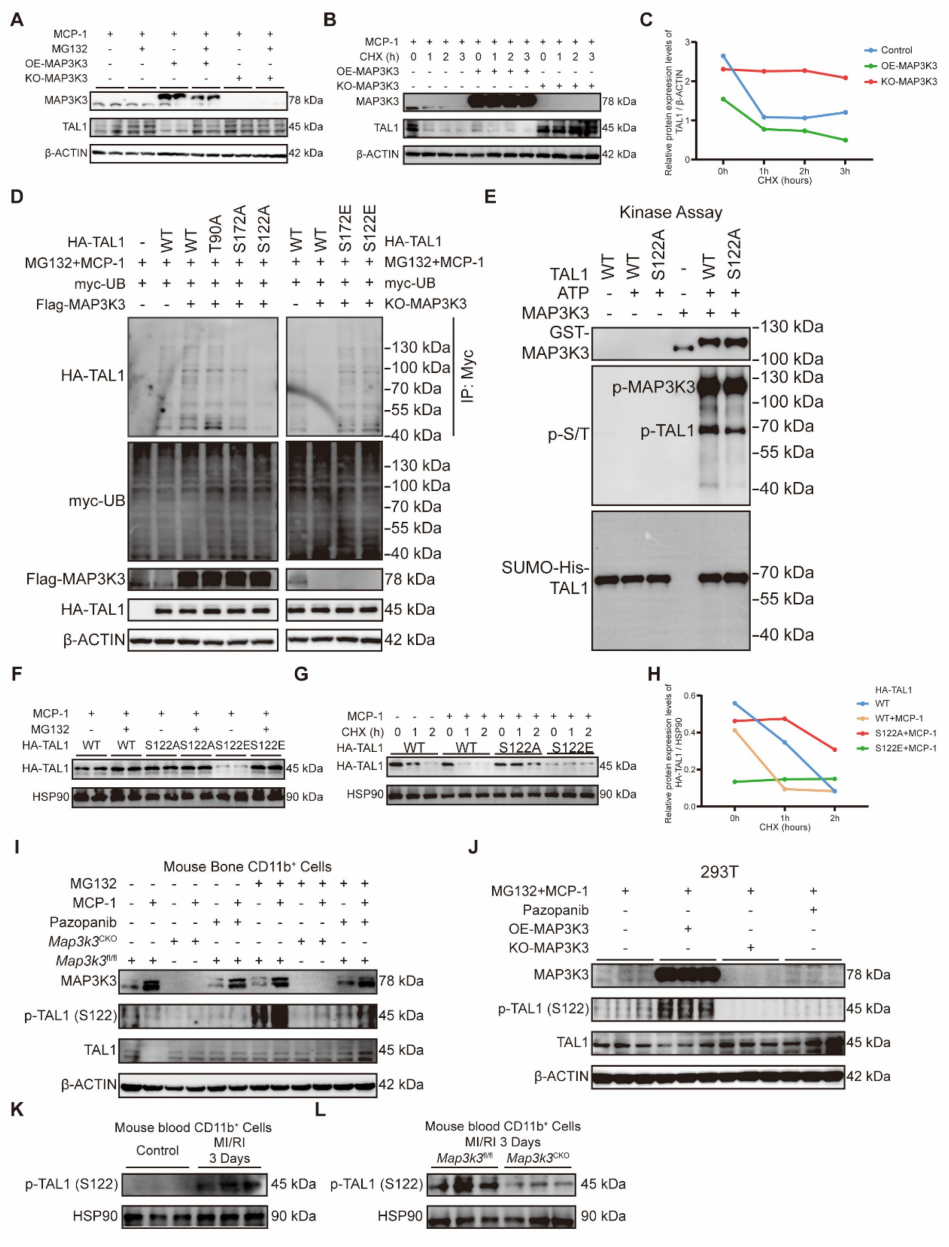
** MAP3K3 induced the proteasome-dependent degradation of TAL1 by phosphorylation on Ser-122. (A)** Western blots of MAP3K3 and TAL1 proteins from 293T treated with MCP-1 and MG132 or not, transfected with MAP3K3 overexpression plasmid and MAP3K3 knockdown adenovirus or not. **(B)** Western blots of MAP3K3 and TAL1 proteins from 293T treated with MCP-1 and CHX time course, transfected with MAP3K3 overexpression plasmid and MAP3K3 knockdown adenovirus or not. **(C)** Relative protein level of TAL1 transfected with MAP3K3 overexpression plasmid, or MAP3K3 knockdown adenovirus along CHX time course. **(D)** Western blots of HA-TAL1 proteins from IP-myc and myc-UB, Flag-MAP3K3, and HA-TAL1 proteins from input. 293T was treated with MCP-1 and MG132, transfected with myc-UB plasmid, Flag-MAP3K3 plasmid or MAP3K3 knockdown adenovirus, and HA-TAL1 plasmid mutated at WT, T90A, S172A, S122A, S172E, or S122E. **(E)** *In vitro* kinase assay was performed using purified recombinant GST-MAPK3K3 kinase and purified SUMO-his-TAL1 protein (WT or S122A mutant). Protein expression levels of GST-MAP3K3, p-S/T, and SUMO-his-TAL1 were detected by western blots. **(F)** Western blots of HA-TAL1 proteins from 293T treated with MCP-1 and MG132, transfected with HA-TAL1 plasmid mutated at WT, S122A, or S122E. **(G)** Western blots of HA-TAL1 proteins from 293T treated with MCP-1 and CHX time course, transfected with HA-TAL1 plasmid mutated at WT, S122A, or S122E. **(H)** Relative protein level of HA-TAL1 transfected with HA-TAL1 plasmid mutated at WT, S122A, or S122E along CHX time course.** (I)** Western blots of MAP3K3, p-TAL1 (S122), and TAL1 proteins from bone marrow myeloid cells of *Map3k3*^fl/fl^ mice and *Map3k3*^CKO^ mice, treated with MCP-1, MG132, and Pazopanib or not. **(J)** Western blots of MAP3K3, p-TAL1 (S122), and TAL1 proteins from 293T treated with MCP-1, MG132, and Pazopanib or not, transfected with MAP3K3 overexpression plasmid and MAP3K3 knockdown adenovirus or not. **(K)** Western blots of p-TAL1 (S122) proteins from blood myeloid cells of control mice and MI/RI mice. **(L)** Western blots of p-TAL1 (S122) proteins from blood myeloid cells of *Map3k3*^fl/fl^ mice and *Map3k3*^CKO^ mice after MI/RI.

As we found above that the regulation of MAP3K3 on F11r was in a phosphorylation-dependent manner, we attempted to unveil whether TAL1 can be phosphorylated by MAP3K3. Previous studies on TAL1 all showed that TAL1 can be ubiquitinated after being phosphorylated on three highly conserved residues, Ser-122 41, Ser-172 42, or Thr-90 43. We first tested whether the decrease of TAL1 by MAP3K3 might be due to protein degradation. Proteasome inhibitor MG132 successfully decreased the degradation of TAL1 due to the overexpression of MAP3K3 (Figure 6A), while using cycloheximide (CHX) to suppress protein synthesis showed that the degradation of TAL1 protein was accelerated after overexpression of MAP3K3 and the stability of TAL1 protein increased after knockout of MAP3K3 (Figure 6B-C). These results supported that MAP3K3 reduces TAL1 by inducing its proteasome-dependent degradation.

To determine which site on TAL1 was phosphorylated by MAP3K3, we predicted the kinase-specific phosphorylation sites in proteins on GPS 6.0 44. MAP3K3 and its downstream kinases were predicted to phosphorylate TAL1 on Ser-122 and Ser-172 at the highest score (Table S1). After comprehensive consideration of previous studies and our prediction results, we mutated Ser-122, Ser-172, or Thr-90 of TAL1 to Alanine (A). Overexpression of MAP3K3 increased the phosphorylation of TAL1 detected by pan phosphorylated antibody and increased the ubiquitination of TAL1. T90A mutation failed to decrease the phosphorylation and ubiquitination, while S172A mutation and S122A mutation decreased the phosphorylation and ubiquitination of TAL1 significantly. We further respectively mutated Ser-122 or Ser-172 to Glutamic acid (E) to mimic phosphorylation. Knockout of MAP3K3 reduced the phosphorylation and ubiquitination of TAL1 while S122E or S172E mutation maintained the ubiquitination of TAL1 (Figure 6D). To determine the direct phosphorylation function of MAP3K3 on TAL1, we performed an *in vitro* kinase assay and found that S122 could be phosphorylated directly by MAP3K3 (Figure 6E).

S122A mutation decreased the degradation of TAL1, while S122E mutation accelerated this process which can be reversed by MG132 (Figure 6F). Using CHX to suppress protein synthesis showed that the degradation of TAL1 protein was accelerated after S122E mutation and the stability of TAL1 protein increased after S122A mutation (Figure 6G-H). Bone marrow myeloid cells stimulated by monocyte chemokine MCP-1 can cause the increase of MAP3K3 and the degradation of TAL1 while using MG132 can help the observation of phosphorylation of TAL1 at Ser-122. Cells from *Map3k3*^CKO^ mice or treated with pazopanib showed lower phosphorylation and higher stability of TAL1 (Figure 6I). The same phenomenon was also observed in 293T cell lines (Figure 6J). In myeloid cells from the blood of MI/RI mice, we also observed increasing expression levels of p-TAL1 (S122) (Figure 6K and Figure S7B), while in *Map3k3*^CKO^ mice, this expression status was reversed (Figure 6L and Figure S7C).

### Pazopanib ameliorated MI/RI by decreasing phosphorylation function of MAP3K3 and diapedesis from bone

**Figure 7:**
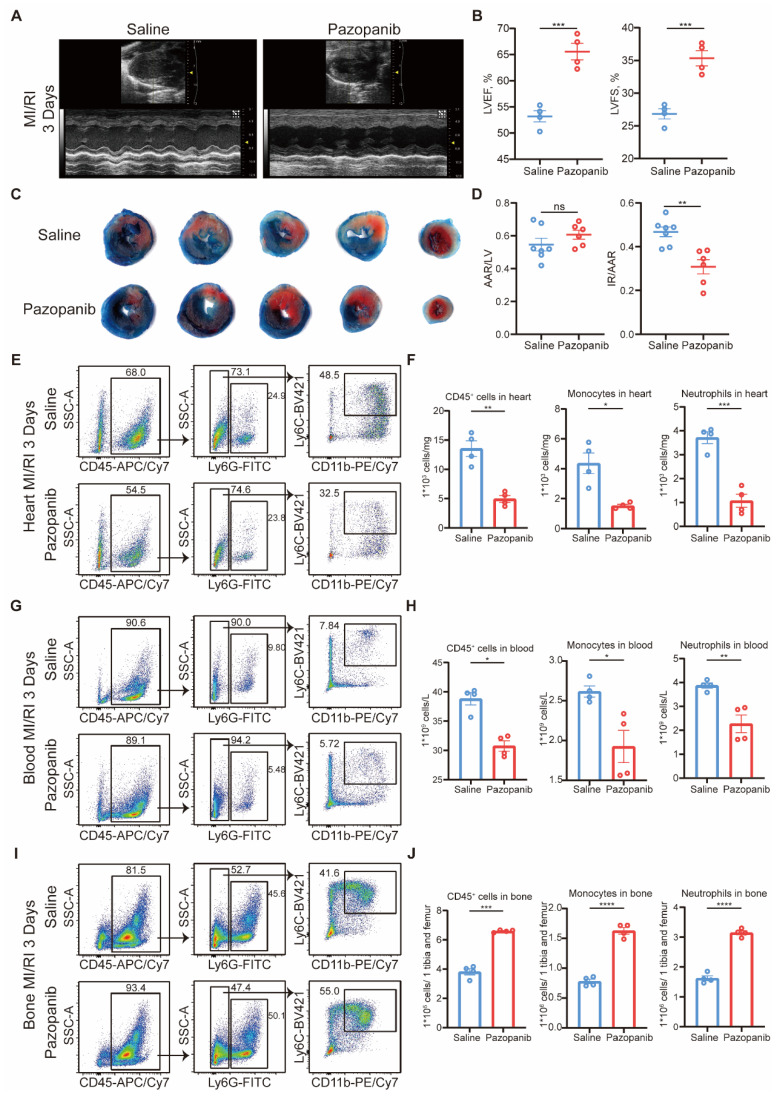
** Pazopanib ameliorated MI/RI by decreasing phosphorylation function of MAP3K3 and diapedesis from bone. (A)** Representative images of echocardiography of saline treated mice and Pazopanib treated mice undergoing MI/RI. **(B)** LVEF (left) and LVFS (right, unpaired *t* test for both) of MI/RI + Saline mice (*n*=4), and MI/RI + Pazopanib mice (*n*=4). **(C)** Representative image of left ventricular tissue sections stained with Evans blue and 2,3,5-triphenyl tetrazolium chloride at 3 days after MI/RI to delineate the area at risk (AAR, red) and the infarcted area (IR, white). **(D)** The ratios of AAR/LV (left) and IR/AAR (right, unpaired *t* test for both) were compared (*n*=7 for MI/RI + Saline mice, *n*=6 for MI/RI + Pazopanib mice). **(E)** Representative flowcytometric dot plot analysis of infiltrating myeloid cells in heart of saline treated mice and Pazopanib treated mice. **(F)** Proportion of leukocytes (left, unpaired *t* test), monocytes (middle, unpaired *t* test with Welch's correction), and neutrophils (right, unpaired *t* test) in heart of MI/RI + Saline mice (*n*=4), and MI/RI + Pazopanib mice (*n*=4). **(G)** Representative flowcytometric dot plot analysis of myeloid cells in blood of saline treated mice and Pazopanib treated mice. **(H)** Proportion of leukocytes (left, Mann-Whitney test), monocytes (middle, unpaired *t* test), and neutrophils (right, unpaired *t* test) in blood of MI/RI + Saline mice (*n*=4), and MI/RI + Pazopanib mice (*n*=4).** (I)** Representative flowcytometric dot plot analysis of myeloid cells in bone of saline treated mice and Pazopanib treated mice. **(J)** Proportion of leukocytes (left, unpaired *t* test with Welch's correction), monocytes (middle, unpaired *t* test), and neutrophils (right, unpaired *t* test) in bone of MI/RI + Saline mice (*n*=4), and MI/RI + Pazopanib mice (*n*=4). All data were displayed as mean ± SEM. ns, *P* > 0.05; * *P* < 0.05; ** *P* < 0.01; *** *P* < 0.001; **** *P* < 0.0001.

The above findings suggested that MAP3K3 and its kinase activity would be a potential therapeutic target for treating MI/RI and other inflammatory diseases related to diapedesis. Pazopanib has shown curative effect in acute lung injury 16, and effective inhibition of diapedesis *in vitro*. Thus, we tested the effects of pazopanib in MI/RI. Pazopanib treatment led to amelioration of MI/RI reflected in elevated LVEF and LVFS (Figure 7A-B), reduced infarcted size (Figure 7C-D), and reduced apoptosis of cardiomyocyte stained by TUNEL (Figure S8A).

Flowcytometric dot plot analysis on MI/RI mice showed that pazopanib treatment decreased proportion of both neutrophils and monocytes diapedesis to heart (Figure 7E-F) and blood (Figure 7G-H) from bone (Figure 7I-J). The *in vivo* infiltration experiment also showed that myeloid cells treated with pazopanib decreased diapedesis to the peritoneum after TG injection (Figure S8B). HE staining also showed less diapedesis and disorganization in the heart after MI/RI with pazopanib treatment (Figure S8C). Immunofluorescence staining of MI/RI tissue sections also showed that after pazopanib treatment, myeloid cells tended to adhere to the blood vessels instead of diapedesis through blood vessels, resulting in the decrease of infiltrating myeloid cells in heart tissue (Figure S8D).

Thus, we found pazopanib as an excellent treatment to ameliorate MI/RI by decreasing the phosphorylation function of MAP3K3 and diapedesis.

## Discussion

Leukocyte diapedesis plays a central role in inflammatory diseases. In the context of MI/RI, we disclosed the essential role of MAP3K3 in myeloid cell diapedesis. The high expression level of MAP3K3 in peripheral blood myeloid cells of MI/RI patients and its relationship with cardiac markers confirmed the important role of MAP3K3 in the MI/RI process. However, a larger clinical validation set is needed to further confirm this finding. Myeloid cell-specific deficiency of *Map3k3* ameliorated MI/RI by inhibiting myeloid cell diapedesis from the bone. We expanded our research to a broader range of inflammatory models, such as LPS-induced sepsis, and found that MAP3K3 deficiency also led to impaired diapedesis. *In vivo* peritonitis experiments further confirmed the critical role of MAP3K3 in diapedesis.

Among the process of leukocyte diapedesis, the mechanisms of leukocyte chemotaxis 4 and leukocyte adhesion to vessels 6 have been studied extensively, while the processes governing leukocyte transmigration through vessels, particularly the upstream regulatory pathways, remain poorly understood. RNA-seq on monocytes found that depletion of MAP3K3 led to decreased *F11r* and increased adhesion-related genes *Itgb3*, *Adgre4*, and *Itgax*. As JAM-A showed an important function of integrin internalization and de-adhesion of leukocyte to endothelial cell, we considered MAP3K3 might influence myeloid cell transmigration through vessels by regulating *F11r* expression. Further exploring the potential transcription factors on *F11r*, we focused on an inhibitory transcription factor, TAL1, and confirmed its inhibitory effect on *F11r* transcription. JAM-A expression changed in the same direction as MAP3K3 while altering TAL1 expression in the opposite direction was able to reverse changes in JAM-A. To determine whether the phenotypes that we observed here are dependent on the kinase activity of MAP3K3, we utilized pazopanib, a known MAP3K3 kinase inhibitor, and observed similar changes in TAL1 and JAM-A expression. *In vitro* transwell assay and adhesion assay confirmed the role of MAP3K3/JAM-A in the de-adhesion and transmigration of myeloid cells, and the regulation of integrin internalization by MAP3K3/JAM-A was also verified. For the first time, we identified the MAP3K3/TAL1/JAM-A regulatory pathway as a key mechanism controlling myeloid cell transmigration through blood vessels. Due to the important role of JAM-A on platelets and the discovery of JAM-A on myeloid cells in our study, the interaction of JAM-A between different cell types is a topic worth exploring in the future. To be noticed, although decreasing myeloid cell transmigration could decrease inflammation in target tissue, inflammation on the blood vessels, such as atherosclerosis, might aggravate 29. Therefore, more precise tissue- and time- treatments are required for complex diseases such as MI/RI with atherosclerosis. For instance, a short-term intervention immediately post-MI. Alternatively, developing delivery systems (e.g., nanoparticles activated by myeloid cell-specific enzymes) that preferentially target the infarct zone could spare the systemic vasculature. Meanwhile, combined adjunctive plaque-stabilizing therapy, such as high-intensity statins or novel anti-inflammatory drugs like colchicine and Firsekibart, could counteract any potential pro-inflammatory effects on the vasculature, resulting in a synergistic therapeutic outcome.

Since the phenotypes are dependent on the kinase activity of MAP3K3, we further explored the phosphorylation and ubiquitination of TAL1. Based on a combined analysis of previous research and our GPS6.0 prediction results, we identified Ser-122, Ser-172, and Thr-90 as potential phosphorylated sites on TAL1. *In vitro* kinase assays confirmed that MAP3K3 can directly phosphorylate TAL1 at Ser-122, which is a new substrate of MAP3K3 kinase. In addition, mutation at Ser-122 resulted in the most pronounced change in ubiquitination.

Given that MAP3K3 showed an important regulatory function in MI/RI through myeloid diapedesis in a phosphorylation-dependent manner, we administrated pazopanib to treat MI/RI for potential future clinical transformation. As an anti-tumor drug, the cardiotoxicity of pazopanib (potential QT prolongation and heart failure) has been the focus of previous research 45, 46. However, our study found that strictly controlled use of pazopanib (low-dose, 1.5 mg/kg vs. >30 mg/kg, and short-term administration, 3 days vs. long-term) improved MI/RI by decreasing myeloid diapedesis, and weakening the inflammatory response. Future basic and clinical studies may help to determine the optimal dosage and timing of pazopanib administration more precisely, as well as possible remedial measures 45 (such as Bisoprolol fumarate, spironolactone, furosemide, and ramipril, which are commonly used treatments after PCI), to better exert pazopanib 's anti-inflammatory effects and reduce its cardiac toxicity. In addition, XJ-8, a natural compound isolated from Sanguis draxonis, is reported to inhibit MAP3K3 function in platelets 18, and whether it could inhibit diapedesis is worth further research. MicroRNA-145 47 and miR-124-3p 48 are also reported to regulate MAP3K3 function, which might also be potential targets to treat MI/RI.

## Conclusion

In conclusion, we revealed the MAP3K3/TAL1/JAM-A pathway as an important regulator of myeloid cell diapedesis. Inhibiting this pathway can decrease infiltrating myeloid cells in inflammatory tissue and ameliorate injuries, such as MI/RI and sepsis. The following in-depth mechanism study showed that MAP3K3 could phosphorylate TAL1 at Ser-122, which triggered the ubiquitination of TAL1 and decreased its inhibitory transcription function on *F11r*. Through JAM-A, MAP3K3 regulated integrin internalization and facilitated de-adhesion and transmigration of myeloid cells. As a kinase inhibitor of MAP3K3, pazopanib inhibited JAM-A expression and myeloid cell diapedesis, thus ameliorating MI/RI. Pazopanib could be a potential treatment for excessive inflammation during MI/RI or other inflammatory diseases.

## Supplementary Material

Supplementary methods, figures and tables.

## Figures and Tables

**Table 1:** Clinical characteristics of 66 patients with MI/RI and 6 Control group donors.

Characteristics	MI/RI (*n*=66)	Control (*n*=6)	*P* value
Age	67.23±1.35	58.00±4.06	0.051
Sex, male (%)	42 (63.6)	5 (83.3)	0.332
Hypertension (%)	42 (63.6)	5 (84.4)	0.332
Diabetes (%)	32 (48.5)	3 (50.0)	0.943
Hyperlipidemia (%)	15 (33.7)	2 (66.7)	0.558
Stroke (%)	7 (10.6)	0 (0.0)	0.401
STEMI (%)	31 (47.0)		
Degree of stenosis in the culprit vessel (%)	93.56±1.05	25.00±8.06	<0.0001
Ejection fraction (%)	53.66±1.26	59.67±3.08	0.145
cTnT (ng/mL)	1.41±0.25	0.01±0.00	<0.0001
CK-MB (U/L)	27.83±3.11	15.33±1.26	0.0004
Pro-BNP (pg/mL)	2775.31±695.63	101.07±30.17	<0.0001
LDH (U/L)	392.79±41.24	169.33±9.07	0.0017
WBC count (10^9^/L)	8.48±0.34	6.48±1.14	0.1262
Neutrophil percentage (%)	68.82±1.01	57.55±3.31	0.002
Lymphocyte percentage (%)	20.53±0.93	30.20±3.03	0.004
NLR%	4.10±0.31	2.07±0.34	0.043
Monocyte percentage (%)	8.56±0.27	7.68±0.44	0.331
Neutrophil count (10^9^/L)	5.91±0.31	3.82±0.74	0.049
Lymphocyte count (10^9^/L)	1.63±0.83	2.66±0.70	0.135
NLR	4.11±0.32	1.85±0.33	0.001
Monocyte count (10^9^/L)	0.77±0.60	0.49±0.80	0.069
Glycosylated hemoglobin (%)	6.84±0.25	5.82±0.25	0.108
CRP (mg/L)	21.12±3.40	0.97±0.33	<0.0001
Previous MI episodes (%)	11 (16.7)	0 (0.0)	
Dual-antiplatelet therapy, aspirin + clopidogrel or ticagrelor (%)	23 (34.8)		
Statins (%)	66 (100)		
β-blocker (%)	40 (60.6)	2 (66.7)	0.193
Anti-hypertension therapy (%)	42 (63.6)	5 (84.4)	0.332
Anti-diabetes therapy (%)	32 (48.5)	3 (50.0)	0.943

Data presented as mean ± standard error of the mean (SEM) or number (percentage). Statistical analysis was described in methods. STEMI, ST-elevated myocardial infarction; cTnT, cardiac troponin T; CK-MB, creatine kinase MB isoenzyme; pro-BNP, pro B-type natriuretic peptide; LDH, lactate dehydrogenase; WBC, white blood cell; NLR, neutrophil-to-lymphocyte ratio; CRP, C-reactive protein.

## Abbreviations

MI: myocardial infarction
MI/RI: myocardial ischemia/reperfusion injury
ACS: acute coronary syndrome
STEMI: ST-elevated myocardial infarction
CAD: coronary artery disease
ICM: ischemic cardiomyopathy
MAP3K3: mitogen-activated protein kinase kinase kinase 3
TAL1: T cell acute lymphocytic leukemia
PBMCs: peripheral blood mononuclear cells
MCP-1: chemoattractant protein 1
JAM-A: junctional adhesion molecule-A
CK-MB: creatine kinase M-type
cTnT: cardiac troponin T
LDH: lactate dehydrogenase
pro-BNP: pro B-type natriuretic peptide
CRP: C-reactive protein
IL-6: interleukin-6
WBC: white blood cell
NLR: neutrophil-lymphocyte-ratio
LVEF: left ventricular ejection fraction
LVFS: left ventricular fraction shortening
TUNEL: TdT-mediated dUTP nick end labeling
LPS: Lipopolysaccharide
TG: thioglycolate
GO: gene ontology
KEGG: Kyoto Encyclopedia of Genes and Genomes
Ser: serine
Thr: threonine
CHX: cycloheximide
