# Supplementary material for: MAP3K3 Contributes to Myocardial Ischemia/Reperfusion Injury by Promoting Myeloid Cell Diapedesis through TAL1/JAM-A Pathway
Source: Theranostics. 2026 Jan 1;16(4):1959–74. doi: 10.7150/thno.122130 (PMC12680592; doi:10.7150/thno.122130)
Supplement: Supplementary file 1 — Supplementary methods, figures and tables. [file thnov16p1959s1.pdf]

SUPPLEMENTARY MATERIAL

MAP3K3 Contributes to Myocardial Ischemia/Reperfusion Injury by Promoting Myeloid Cell Diapedesis through TAL1/JAM-A Pathway

Shiyu Hu[1,3,4,5,6,*], Jian Zhang[1,3,4,5,6,*], Jingpu Wang[1,3,4,5,6], Chenguang Li[1,3,4,5,6], Yiwen Wang[1,3,4,5,6], Jiayu Liang[1,3,4,5,6], Rong Huang[1,3,4,5,6], Ji'e Yang[7], Yang Gao[1,3,4,5,6], Yanan Qu[1,3,4,5,6], Hongbo Yang[1,3,4,5,6], Juying Qian[1,3,4,5,6], Wenwen Tang[2,✉], Jiatian Cao[1,3,4,5,6,✉], Feng Zhang[1,3,4,5,6,✉], Junbo Ge[1,3,4,5,6]

Supplementary Material

Supplementary Methods

Figure S1 to S8

Table S1 to S2

Supplementary Methods

Detailed information of reagents and antibodies is provided in Table S2.

Patients

Peripheral blood of the MI/RI group was obtained 12 hours (to better reflect the changes during the acute inflammatory phase, especially at the peak period of diapedesis of myeloid cells [1-3]) after PCI in 66 patients diagnosed with acute coronary syndrome (ACS) (clear diagnosis of myocardial infarction, STEMI and non-STEMI, and the presence of culprit vessels, stenosis greater than 90%, on coronary angiography). Peripheral blood of the control group was obtained from 6 donors with negative coronary angiography results. The control group was defined as angiographic stenosis <50% in all epicardial vessels. These patients had no clinical or biochemical evidence of acute ischemia (normal high-sensitivity troponin without dynamic change; no ischemic ECG changes) and no ACS within the preceding 3 months. This group was selected to provide a stable, non-ischemic comparator in which severe ischemia-driven myeloid mobilization is not expected [4].

Human sample collection

EDTA anticoagulant tubes were used to collect blood. All blood samples were transported at 4 °C within 1 hour and isolated into neutrophils or monocytes.

Quantitative real-time PCR

Trizol was used to extract total mRNA and a reverse transcription kit was used to reversely transcribe mRNA to cDNA. A qPCR Mix was used to perform real-time quantitative PCR (RT-qPCR). Data analysis was performed following the comparative CT ($2^{-\Delta\Delta CT}$) method [5]. The sequences of RT-qPCR primers for human *MAP3K3* were: Forward Primer, CAGGTGCGGATCAAGGCTT; Reverse Primer, CCGCTCAGGAACATAGCCAG. The sequences of real-time qPCR primers for human *IL6* were: Forward Primer, CCTGAACCTTCCAAAGATGGC; Reverse Primer, TTCACCAGGCAAGTCTCCTCA.

Animals study design and establishment of MI/RI models

Mouse MI/RI model was constructed with a 45-minute ligation time at the left anterior descending coronary artery [6].

The *Map3k3*$^{fl/fl}$ and *Map3k3*^{CKO} mice were constructed by Cyagen Biosciences in C57BL/6 mice (*Lyz2*-Cre mice were from Jackson Laboratory Bar, Harbor, ME). Littermate mice with average age of 6-8 weeks were used for all experiments.

Sex-matched wild-type (WT) mice (Shanghai Jiesijie) aged 6-8 weeks were randomly divided into the saline group (200 μL saline) and pazopanib-treated group. Pazopanib-treated mice were injected intraperitoneally with 1.5 mg/kg pazopanib per mouse as previously described [7, 8]. The mice were injected immediately after surgery (after ligation, before reperfusion), 24 hours after surgery, and 48 hours after surgery.

Mouse blood CD11b$^+$ cells were isolated from mouse blood.

Echocardiography

Transthoracic echocardiography was conducted using M-mode tracing recordings in VeVo 2100 Imaging System (VisualSonics, Toronto, ON, Canada) in mice 3 days after MI/RI surgery as previously described [2].

TTC-Evans blue analysis

TTC-Evans blue analysis was conducted as previously described [2]. The infarct area was not stained by TTC or Evans blue (white), the area at risk (AAR) was stained by TTC (red), and the non-ischemic area was stained by Evans blue (blue).

Flow cytometry

Flow cytometry was conducted as previously described [2, 9]. For mice undergoing MI/RI, 1 day, 3 days, or 5 days after surgery, or mice undergoing sepsis, hearts, lungs, and spleens of mice were harvested for flow cytometry. EDTA anticoagulant tubes were used to collect the blood of mice. The bone marrow cells were washed from the femurs and tibias of mice.

For bone marrow myeloid cells, after experiments, extracellular staining was conducted with or without fixation and permeabilization.

All agents and antibodies used are provided in Table S2.

The cell suspension was acquired on BD FACSAriaIII CellSorting System (BD Bio), and the data were analyzed by FlowJo v.9.5.2 software.

Immunofluorescence analysis and HE staining of mice heart sections

The 3 day-post MI/RI mice heart slices were prepared as previously described [2].

DAPI was used for nucleus staining. Primary antibodies used for immunofluorescence analysis were: anti-Ly6G antibody, anti-Ly6C antibody, anti-CD31 antibody, anti-MAP3K3 antibody, and anti-cTnT antibody.

TUNEL staining was performed with In Situ Cell Death Detection Kit-Fluorescein.

All agents and antibodies used are provided in Table S2.

Hematoxylin-eosin staining was conducted for the analysis of inflammatory cell infiltration.

The stained slides were photographed with a confocal microscope (Olympus FV3000, Japan).

Bone marrow myeloid cells isolation and stimulation

Bone marrow myeloid cells were isolated from *Map3k3*$^{fl/fl}$ and *Map3k3*^{CKO} mice aged 6–8 weeks. Briefly, the mouse was sacrificed by decapitation, and the femurs and tibias were carefully isolated. After flushing the bone marrow out with PBS, the cell suspension was filtered through a 70 μm filter and centrifuged at 1500 rpm for 10 minutes. The precipitate was resuspended to isolate myeloid cells.

Myeloid cells were treated with MCP-1 [10], WKYMVM [11], or pazopanib [7], and the concentration was determined by concentration gradient experiment in Figure S5G-I.

Analysis of myeloid cell recruitment *in vivo*

This experiment was conducted as previously reported [12]. Briefly, the isolated bone marrow myeloid cells were treated as specified in the Figure legends and labeled with either 1 mM CFSE or 1mM Far-Red DDAO SE in HBSS without Ca^{2+}/Mg^{2+}. After 15 minutes of incubation at 37 °C for labeling, cells were washed twice with HBSS without Ca^{2+}/Mg^{2+}. These two dyes-labeled neutrophils were mixed in a 1:1 ratio and about 10 million total cells were injected i.v. or i.p. into WT recipient mice, which had previously received an intraperitoneal injection of 1.5 mL of 3% thioglycolate 1.5 hours prior. The mice were then euthanized 2.5 hours later, and cells in the peritoneum and blood were collected and analyzed through cell counting and flow cytometry.

RNA Sequencing

RNA sequencing was conducted as previously described [2] by OE Biotech Co. Ltd. (Shanghai, China). The threshold for significantly differential expression genes (DEGs) was set as Q value < 0.05 and fold change > 2 or < 0.5.

Phosphoproteomic analysis

Frozen samples (100 mg) were ground in liquid nitrogen, homogenized in phenol extraction buffer, and mixed with Tris-HCl-saturated phenol. After centrifugation, the phenolic phase was combined with ammonium acetate-methanol, precipitated overnight at −20 °C, and washed with methanol and acetone. The pellet was resuspended in lysate solution, and protein concentration was quantified via bicinchoninic acid assay. For digestion, proteins were reduced with DTT, alkylated with iodoacetamide, and precipitated with acetone. The pellet was enzymatically digested (trypsin, 37 °C, 12 h), and peptides were desalted using C18 cartridges. The phosphoproteomic analysis was conducted by OE Biotech Co. Ltd. (Shanghai, China).

Phosphorylated peptides were enriched using IMAC, eluted, and dried. LC-MS analysis was performed on a timsTOF Pro 2 mass spectrometer with a 60 min gradient. Data were acquired in ion mobility mode (100-1700 m/z) and searched against the Mus musculus database using Spectronaut Pulsar^{TM}, with fixed/variable modifications and a 1% FDR threshold.

Differentially expressed phosphopeptides (fold change > 1.2 or < 0.83, $P < 0.05$) were analyzed for phosphorylation motifs (MOMO), annotated via GO and KEGG, and subjected to protein-protein interaction analysis (STRING).

Dual-Luciferase reporter assay

The Dual-Luciferase reporter assay was conducted as previously described [2] in the 293T cells purchased from ZQXZbio with STR profiling. Briefly, the firefly luciferase plasmids m-*F11r* (Full) or pGL3-Basic (NC) were co-transfected with TAL1 eukaryotic expression vectors (containing m-TAL1 or pcDNA3.1–3*flag) and the pRL-TK renilla luciferase vector (internal reference) into 293T cells. After 48 h, Dual-Luciferase Reporter Assay Kits was used to detect firefly and Renilla luciferase activities in extracted cell lysates. Multifunctional microplate reader (Varioskan^{TM} LUX, Thermofisher) was used to detect the luminescence signal. The experiment was repeated twice.

Transwell assay

TNF-α (20 ng/mL) stimulated bEnd.3 mouse endothelial cell lines were cultured on the insert of 6.5 mm Transwell® with 8.0 μm Pore Polycarbonate Membrane Insert overnight. Isolated bone marrow myeloid cells were suspended in an FBS-free medium. The appropriate volume of RPMI 1640 with FBS, MCP-1 (100 ng/mL), and WKYMVM (5 nM) was added to the lower chamber, and $5*10^4$ cells were suspended in the upper chamber of the 24-well transwell plate. For certain experiments, IgG1 [13], α-JAM-A (20 μg/mL), or pazopanib (20 nM) was added to the lower chamber. For flowcytometric dot plot analysis, cells in the lower chamber after transwell assay were collected and stained. For microscope observation, isolated bone marrow myeloid cells were labeled by 1 mM CellTracker Green CMFDA before the assay, and the bottom of the plate was pre-coated with a poly-L-lysine (0.1 mg/mL) solution for 4 hours. After a

certain time of migration, the cells attached to the bottom were observed by a fluorescence microscope.

Adhesion assay

$1*10^5$ CMFDA -labeled bone marrow myeloid cells were incubated on 48-well culture plates containing TNF-α (20 ng/mL) stimulated endothelial cell monolayers (bEnd.3) for 4 hours. After washing three times with warmed PBS, adherent cells were observed by a fluorescence microscope.

Immunofluorescence staining and confocal microscopy

Bone marrow myeloid cells were attached to Confocal dishes coated by poly-L-lysine (0.1 mg/mL) and then stimulated with MCP-1 (100 ng/mL) and WKYMVM (5 nM). For certain experiments, IgG1, α-JAM-A (20 μg/mL), or pazopanib (20 nM) were added as well. Then, cells were fixed, blocked, permeabilized, and stained (detail of antibodies are provided in Table S2). Images were acquired using a confocal microscope (Olympus FV3000, Japan).

Adenovirus, plasmid, RNA-mediated interference construction and transfection

Adenovirus for knockout of MAP3K3 was purchased from OBiO Technology, and was transfected to 293T cells at MOI=20 for 24 hours.

The mouse plasmids were obtained from Xinzhuo Bio. The MAP3K3 overexpression plasmid was labeled with Flag tag. The WT, S122A, S172A, T90A, S122E, and S172E mutated TAL1 overexpression plasmids were labeled with HA tag. The ubiquitin plasmid was labeled with myc tag. The resulting recombinant plasmids were confirmed by DNA sequencing. The indicated plasmids and empty vectors were transfected into 293T cells using Lipofectamine™ 3000 according to the manufacturer's instructions.

The small interfering RNA (siRNA) targeting human *TAL1* and negative control siRNA were purchased from Xinzhuo Bio. The sequence of siRNA used for human *TAL1* interference was as follows: CAAUGAUAGUAGAGUGAUAtt; UAUCACUCUACUAUCAUUGtt. The siRNAs were then transfected into 293T using Lipofectamine™ 3000 according to the manufacturer's instructions.

Western Blot assay

The Western Blot assay was conducted as previously described [2], and the images were obtained and analyzed using Image Lab 3.0 and ImageJ 1.8.0.

For isolation of plasma membrane proteins, Minute™ Plasma Membrane Protein Isolation and Cell Fractionation Kit (SM-005, Invent Biotechnology) was used according to the manufacturer's instructions.

For protein degradation experiments, MG132 (10 μM for 6 hours before cell collection) and Cycloheximide (CHX) were used.

For Immunoprecipitation (IP) experiments, cells were fully lysed using IP-specific RIPA. The protein was then incubated with the specific magnetic bead-conjugated antibodies at 4 °C overnight. The beads were washed three times using the IP buffer and then added 1* SDS loading buffer. After heating for 5 minutes, the isolated supernatant is immunoprecipitant. Both lysates and immunoprecipitants were examined and analyzed using Western blotting.

All agents and antibodies used are provided in Table S2.

Kinase assay

Recombinant human MAP3K3 protein was incubated with recombinant SUMO-His-tagged TAL1 (WT or mutated to S122A, obtained from HUABio) in the 1* kinase buffer (diluted from 10* kinase buffer), with 250 μM ATP at 30 °C for 30 minutes. The reaction was stopped by adding 1xSDS loading buffer. The samples were boiled for 5 minutes. The proteins were separated by SDS-PAGE and analyzed by Western blotting.

Statistical analysis

Statistical analyses were performed using GraphPad Prism software (version 8.4.2), following previously described methods [2]. For datasets involving two or more independent factors, comparisons were made using two-way ANOVA, while categorical variables were analyzed by chi-square test. A P value of less than 0.05 was considered statistically significant.

References

1. Chia S, Nagurney JT, Brown DF, Raffel OC, Bamberg F, Senatore F, et al. Association of leukocyte and neutrophil counts with infarct size, left ventricular function and outcomes after percutaneous coronary intervention for ST-elevation myocardial infarction. Am J Cardiol. 2009; 103: 333-7.
2. Hu S, Zhang F, Wang J, Zhang J, Li C, Lyu Y, et al. MMP9(High) Neutrophils are Critical Mediators of Neutrophil Extracellular Traps Formation and Myocardial Ischemia/Reperfusion Injury. Adv Sci (Weinh). 2025; 12: e2415205.
3. Rusinkevich V, Huang Y, Chen ZY, Qiang W, Wang YG, Shi YF, et al. Temporal dynamics of immune response following prolonged myocardial ischemia/reperfusion with and without cyclosporine A. Acta Pharmacol Sin. 2019; 40: 1168-83.
4. Rao SV, O'Donoghue ML, Ruel M, Rab T, Tamis-Holland JE, Alexander JH, et al. 2025 ACC/AHA/ACEP/NAEMSP/SCAI Guideline for the Management of Patients With Acute Coronary Syndromes: A Report of the American College of Cardiology/American Heart Association Joint Committee on Clinical Practice Guidelines. Circulation. 2025; 151: e771-e862.
5. Kladi-Skandali A, Sideris DC, Scorilas A. BCL2L12: a multiply spliced gene with independent prognostic significance in breast cancer. Clin Chem Lab Med. 2018; 57: 276-87.
6. Shi H, Gao Y, Dong Z, Yang J, Gao R, Li X, et al. GSDMD-Mediated Cardiomyocyte Pyroptosis Promotes Myocardial I/R Injury. Circ Res. 2021; 129: 383-96.
7. Yuan Q, Basit A, Liang W, Qu R, Luan Y, Ren C, et al. Pazopanib ameliorates acute lung injuries via inhibition of MAP3K2 and MAP3K3. Sci Transl Med. 2021; 13.
8. Kumar R, Knick VB, Rudolph SK, Johnson JH, Crosby RM, Crouthamel MC, et al. Pharmacokinetic-pharmacodynamic correlation from mouse to human with pazopanib, a multikinase angiogenesis inhibitor with potent antitumor and antiangiogenic activity. Mol Cancer Ther. 2007; 6: 2012-21.
9. Jia D, Jiang H, Weng X, Wu J, Bai P, Yang W, et al. Interleukin-35 Promotes Macrophage Survival and Improves Wound Healing After Myocardial Infarction in Mice. Circ Res. 2019; 124: 1323-36.
10. Stamatovic SM, Shakui P, Keep RF, Moore BB, Kunkel SL, Van Rooijen N, et al. Monocyte chemoattractant protein-1 regulation of blood-brain barrier permeability. J Cereb Blood Flow Metab. 2005; 25: 593-606.
11. Kim YE, Park WS, Ahn SY, Sung DK, Sung SI, Kim JH, et al. WKYMVm hexapeptide, a strong formyl peptide receptor 2 agonist, attenuates hyperoxia-induced lung injuries in newborn mice. Sci Rep. 2019; 9: 6815.

12. Zhang N, Tang W, Torres L, Wang X, Ajaj Y, Zhu L, et al. Cell surface RNAs control neutrophil recruitment. Cell. 2024; 187: 846-60 e17.

13. Williams DW, Calderon TM, Lopez L, Carvallo-Torres L, Gaskill PJ, Eugenin EA, et al. Mechanisms of HIV entry into the CNS: increased sensitivity of HIV infected CD14+CD16+ monocytes to CCL2 and key roles of CCR2, JAM-A, and ALCAM in diapedesis. PLoS One. 2013; 8: e69270.

Supplementary Figures and Legends

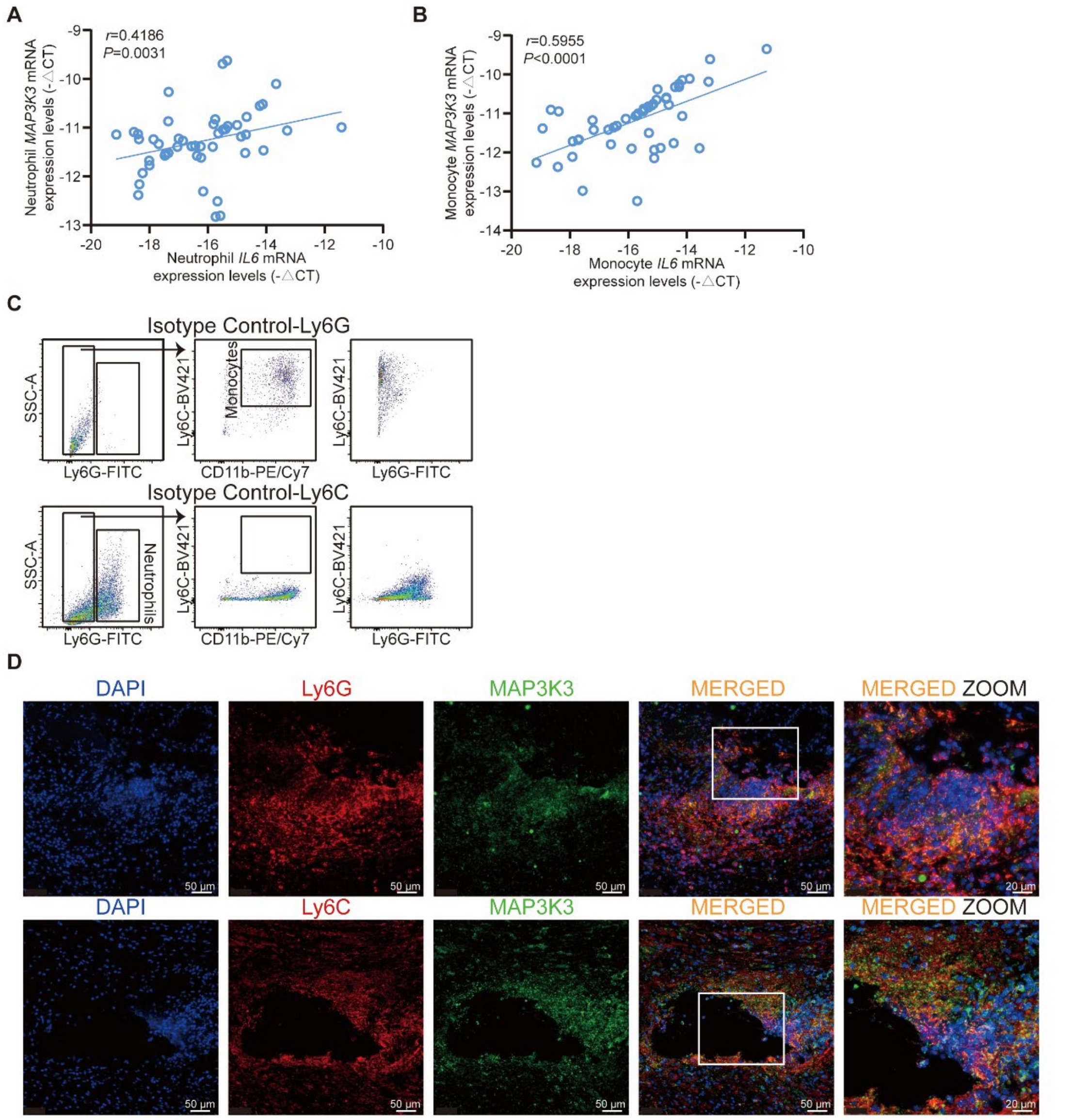

Figure S1. ***MAP3K3*** **expression in myeloid cells increased during MI/RI**

(A) Pearson correlation analysis of *MAP3K3* mRNA levels with *IL6* mRNA levels in neutrophils of patients with MI/RI (*n*=48). **(B)** Pearson correlation analysis of *MAP3K3* mRNA levels with *IL6* mRNA levels in monocytes of patients with MI/RI (*n*=48). **(C)** Isotype control of Ly6G and Ly6C related to flowcytometric dot plot analysis of Figure 1H and Figure 3A. **(D)** Representative fluorescence images of myeloid cells infiltrated in MI/RI tissue sections stained for DAPI (blue), Ly6G/Ly6C (red), MAP3K3 (green), *200, scale bar 50 μm; *400, scale bar 20 μm.

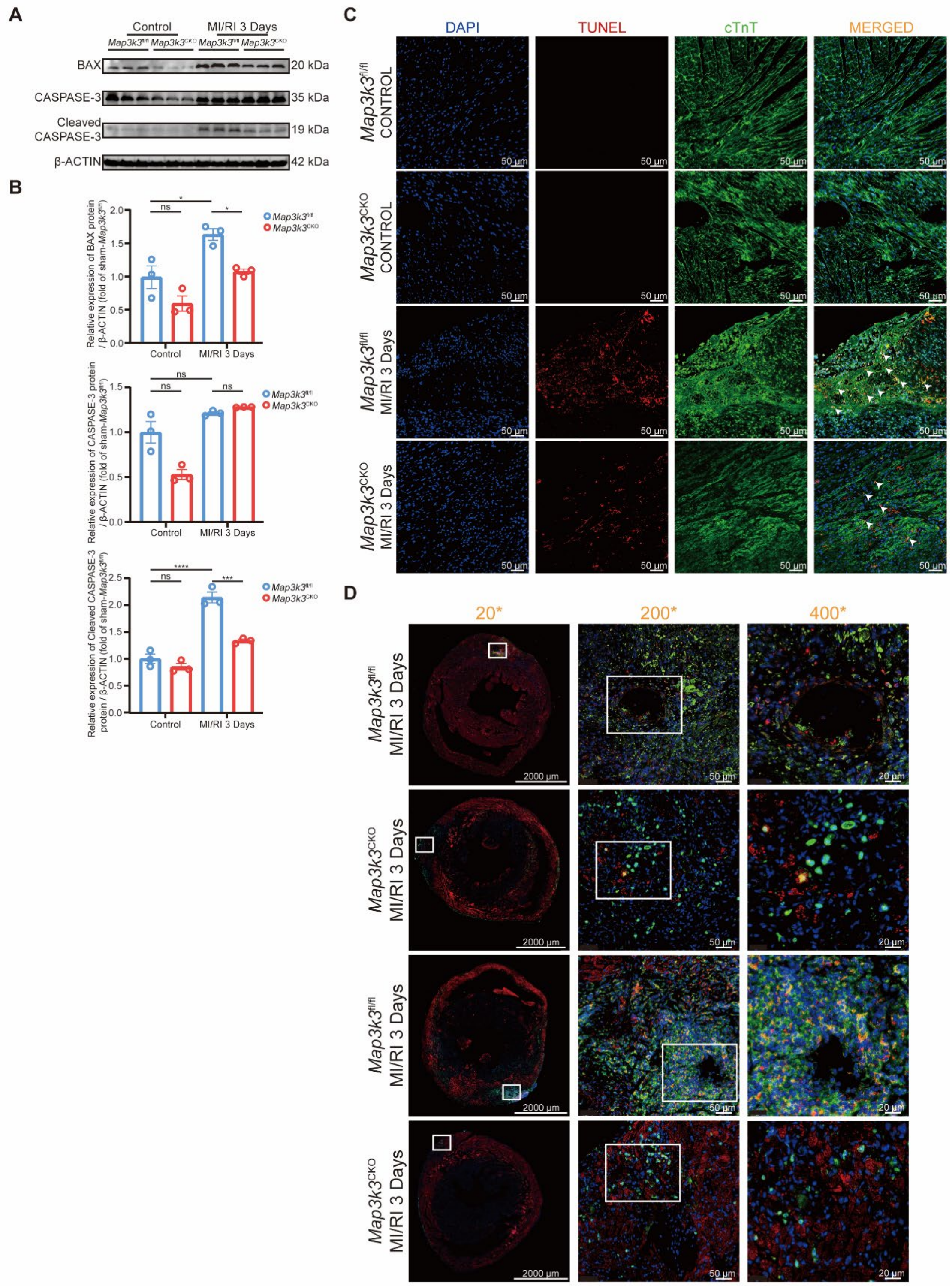

Figure S2. Myeloid-specific *Map3k3* deficiency alleviated MI/RI

(A-B) Relative protein levels of BAX (one-way ANOVA test with Tukey's multiple comparisons test), CASPASE-3 (Kruskal-Wallis's test with Dunn's multiple comparisons test), cleaved CASPAS3-3 (one-way ANOVA test with Tukey's multiple comparisons test) of heart from control + *Map3k3*$^{fl/fl}$ mice, control + *Map3k3*^{CKO} mice, MI/RI + *Map3k3*$^{fl/fl}$ mice, and MI/RI + *Map3k3*^{CKO} mice (n=3 each). **(C)** Representative fluorescence images of apoptotic cardiomyocyte in MI/RI tissue sections stained for

DAPI (blue), TUNEL (red), cTnT (green), *200, scale bar 50 μm. Arrows pointed at apoptotic cells (co-stained area of DAPI and TUNEL). **(D)** Representative fluorescence images of cross-sectional view of the entire heart related to Figure 2E (focusing area framed) stained for DAPI (blue), Ly6G/Ly6C (green), MAP3K3 (red), *20, scale bar 2000 μm; *200, scale bar 50 μm; *400, scale bar 20 μm. All data were displayed as mean ± SEM. ns, $P > 0.05$; * $P < 0.05$; *** $P < 0.001$; **** $P < 0.0001$.

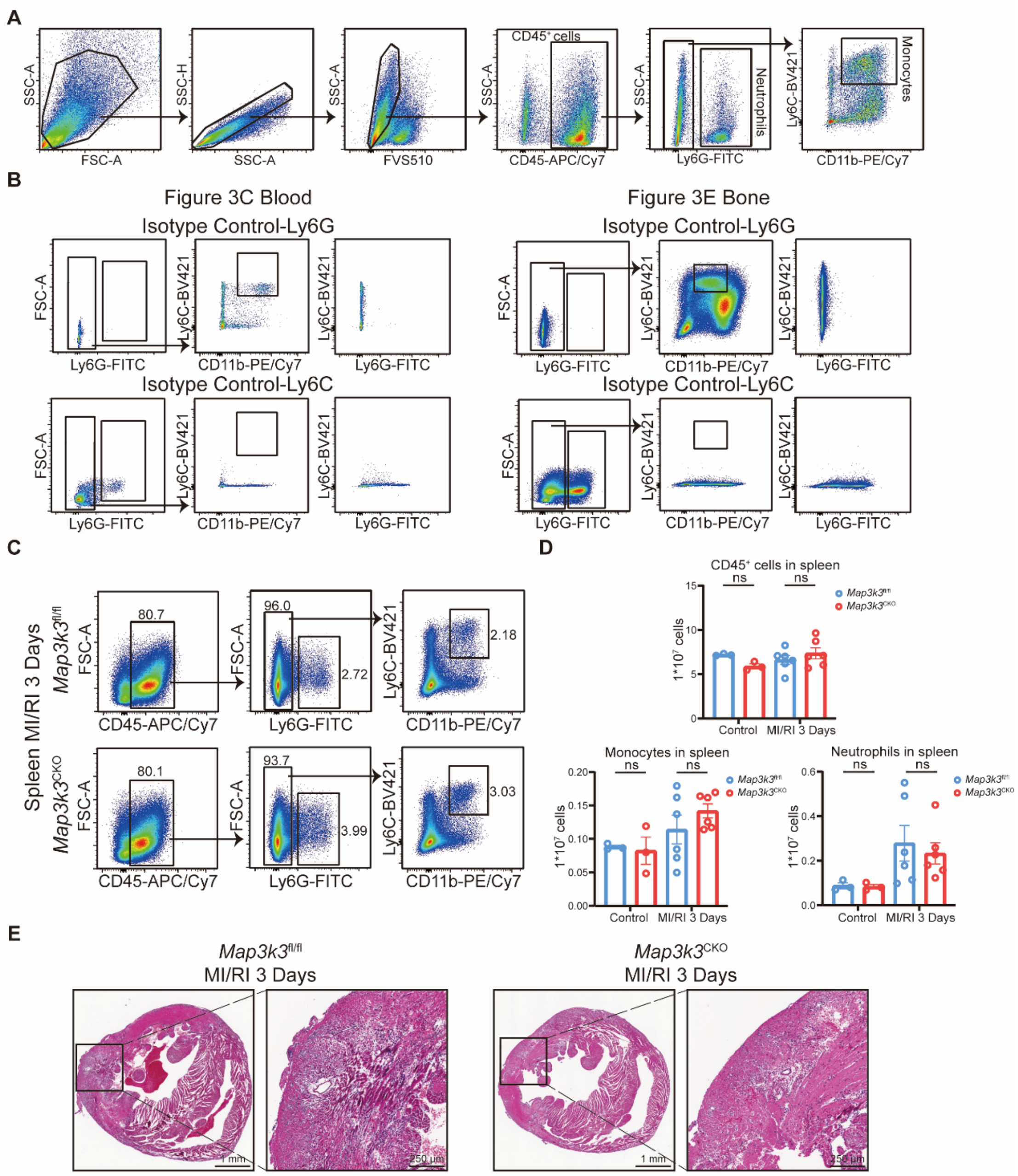

Figure S3. Supplementary data on diapedesis deficiency from bone after MI/RI

(A) Flowcytometric dot plot analysis and staining strategy for neutrophils ($CD45^+Ly6G^+$) and monocytes ($CD45^+Ly6G^-CD11b^+Ly6C^+$) in MI/RI tissues. **(B)** Isotype control of Ly6G and Ly6C related to flowcytometric dot plot analysis of Figure 3C and Figure 3E. **(C)** Representative flowcytometric dot plot analysis of myeloid cells in spleen of *Map3k3*$^{fl/fl}$ mice and *Map3k3*^{CKO} mice. **(D)** Proportion of leukocytes (top, one-way ANOVA test with Tukey's multiple comparisons test), monocytes (bottom left, Brown-Forsythe test with Dunnett's T3 multiple comparisons test), and neutrophils (bottom right, one-way ANOVA test with Tukey's multiple comparisons test) in spleen of control + *Map3k3*$^{fl/fl}$ mice (*n*=4), control + *Map3k3*^{CKO} mice (*n*=4), MI/RI + *Map3k3*$^{fl/fl}$ mice (*n*=6), and MI/RI + *Map3k3*^{CKO} mice (*n*=6). **(E)** Representative HE images of MI/RI tissue sections, *10, scale bar 1 mm; *40, scale bar 250 μm. All data were displayed as mean ± SEM. ns, $P > 0.05$.

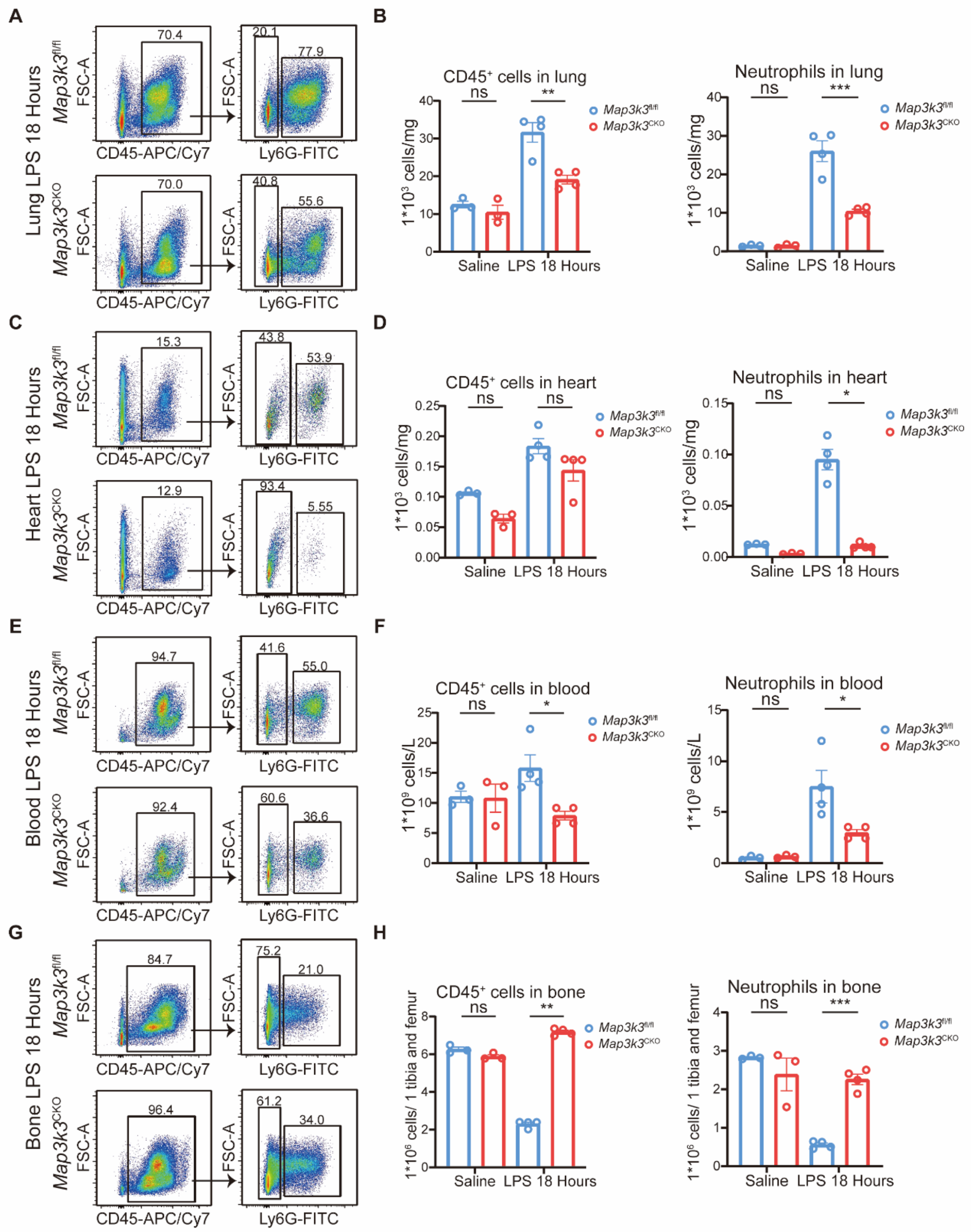

Figure S4. Myeloid-Specific *Map3k3* Deficiency decreased diapedesis from bone after sepsis

(A) Representative flowcytometric dot plot analysis of infiltrating myeloid cells in lung of *Map3k3*$^{fl/fl}$ mice and *Map3k3*^{CKO} mice. **(B)** Proportion of leukocytes (left) and neutrophils (right, one-way ANOVA test with Tukey's multiple comparisons test for both) in lung of saline + *Map3k3*$^{fl/fl}$ mice (n=3), saline + *Map3k3*^{CKO} mice (n=3), LPS + *Map3k3*$^{fl/fl}$ mice (n=4), and LPS + *Map3k3*^{CKO} mice (n=4). **(C)** Representative flowcytometric dot plot analysis of myeloid cells in heart of *Map3k3*$^{fl/fl}$ mice and *Map3k3*^{CKO} mice. **(D)** Proportion of leukocytes (left, Kruskal-Wallis's test with Dunn's multiple comparisons test) and neutrophils (right, Brown-Forsythe test with Dunnett's

T3 multiple comparisons test) in heart of saline + *Map3k3*$^{fl/fl}$ mice (n=3), saline + *Map3k3*^{CKO} mice (n=3), LPS + *Map3k3*$^{fl/fl}$ mice (n=4), and LPS + *Map3k3*^{CKO} mice (n=4). **(E)** Representative flowcytometric dot plot analysis of myeloid cells in blood of *Map3k3*$^{fl/fl}$ mice and *Map3k3*^{CKO} mice. **(F)** Proportion of leukocytes (left, Kruskal-Wallis's test with Dunn's multiple comparisons test) and neutrophils (right, one-way ANOVA test with Tukey's multiple comparisons test) in blood of saline + *Map3k3*$^{fl/fl}$ mice (n=3), saline + *Map3k3*^{CKO} mice (n=3), LPS + *Map3k3*$^{fl/fl}$ mice (n=4), and LPS + *Map3k3*^{CKO} mice (n=4). **(G)** Representative flowcytometric dot plot analysis of myeloid cells in bone of *Map3k3*$^{fl/fl}$ mice and *Map3k3*^{CKO} mice. **(H)** Proportion of leukocytes (left, Kruskal-Wallis's test with Dunn's multiple comparisons test) and neutrophils (right, one-way ANOVA test with Tukey's multiple comparisons test) in bone of saline + *Map3k3*$^{fl/fl}$ mice (n=3), saline + *Map3k3*^{CKO} mice (n=3), LPS + *Map3k3*$^{fl/fl}$ mice (n=4), and LPS + *Map3k3*^{CKO} mice (n=4). All data were displayed as mean ± SEM. ns, $P > 0.05$; * $P < 0.05$; ** $P < 0.01$; *** $P < 0.001$.

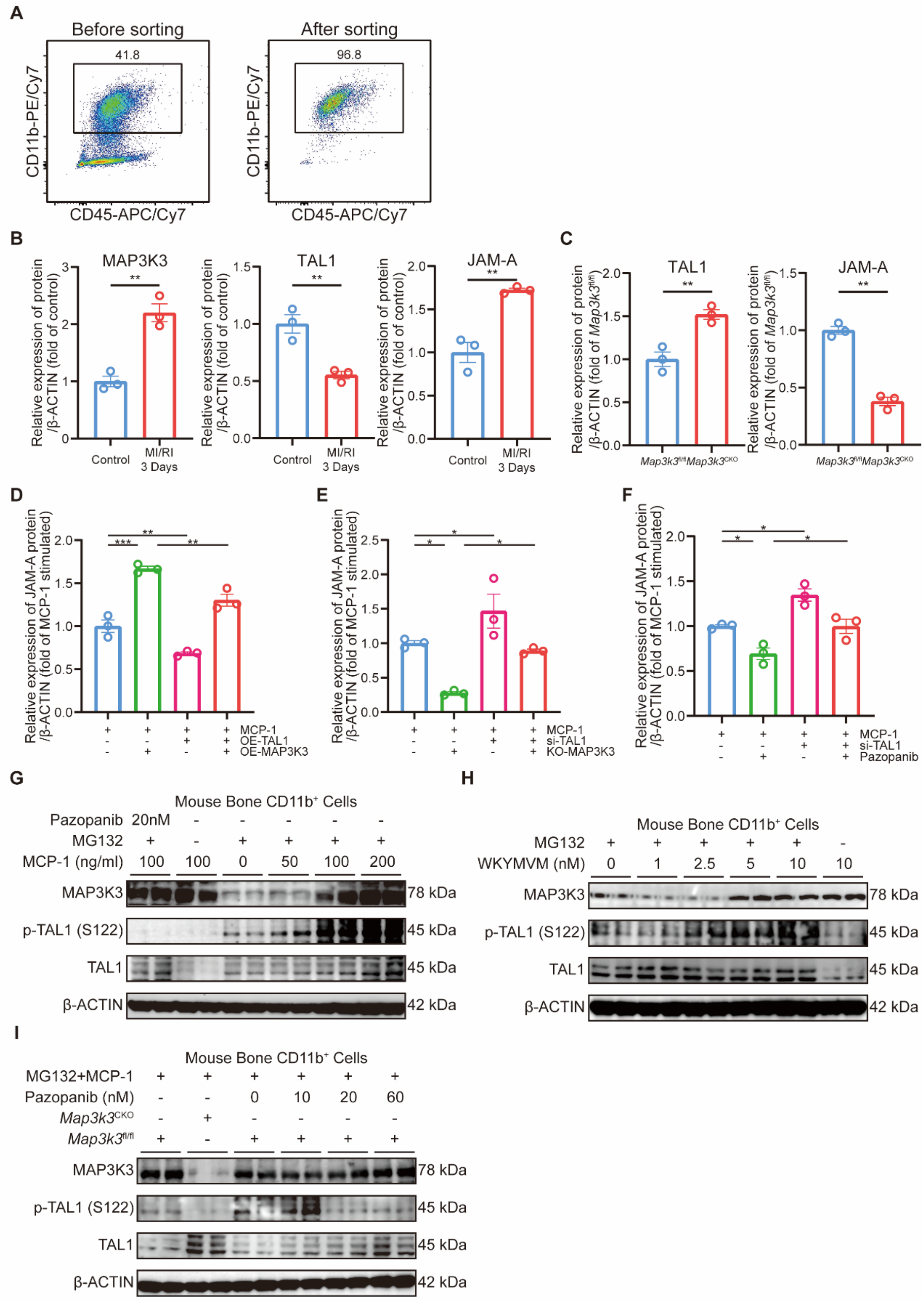

Figure S5. Purity of CD11b sorting, statistical chart on Figure 4, and concentration gradient on MCP-1, WKYMVM, and Pazopanib based on p-TAL1 (S122)

(A) Flowcytometric dot plot analysis for CD11b sorting purity. **(B)** Relative protein levels of MAP3K3, TAL1, and JAM-A of mouse blood myeloid cells from MI/RI mice and control mice, respectively (n=3 each, unpaired t test for all, related to Figure 4H). **(C)** Relative protein levels of TAL1 and JAM-A of mouse blood myeloid cells from

Map3k3$^{fl/fl}$ mice and *Map3k3*^{CKO} mice underwent MI/RI, respectively (n=3 each, unpaired t test for each, related to Figure 4I). **(D)** Relative protein levels of JAM-A from 293T, treated with MCP-1, transfected with TAL1 overexpression plasmid and MAP3K3 overexpression plasmid or not (n=3 each, one-way ANOVA test with Tukey's multiple comparisons test, related to Figure 4L). **(E)** Relative protein levels of JAM-A from 293T, treated with MCP-1, transfected with TAL1 siRNA and MAP3K3 knockdown adenovirus or not (n=3 each, one-way ANOVA test with Tukey's multiple comparisons test, related to Figure 4M). **(F)** Relative protein levels of JAM-A from 293T, treated with MCP-1, transfected with TAL1 siRNA, and Pazopanib or not (n=3 each, one-way ANOVA test with Tukey's multiple comparisons test, related to Figure 4N). **(G)** Western blots of MAP3K3, p-TAL1 (S122), and TAL1 proteins from bone marrow myeloid cells, treated with MCP-1 concentration gradient, Pazopanib, and MG132 or not. **(H)** Western blots of MAP3K3, p-TAL1 (S122), and TAL1 proteins from bone marrow myeloid cells, treated with WKYMVM concentration gradient and MG132. **(I)** Western blots of MAP3K3, p-TAL1 (S122), and TAL1 proteins from bone marrow myeloid cells of *Map3k3*$^{fl/fl}$ mice and *Map3k3*^{CKO} mice, treated with Pazopanib concentration gradient, MCP-1, and MG132 or not. All data were displayed as mean ± SEM. * $P < 0.05$; ** $P < 0.01$; *** $P < 0.001$.

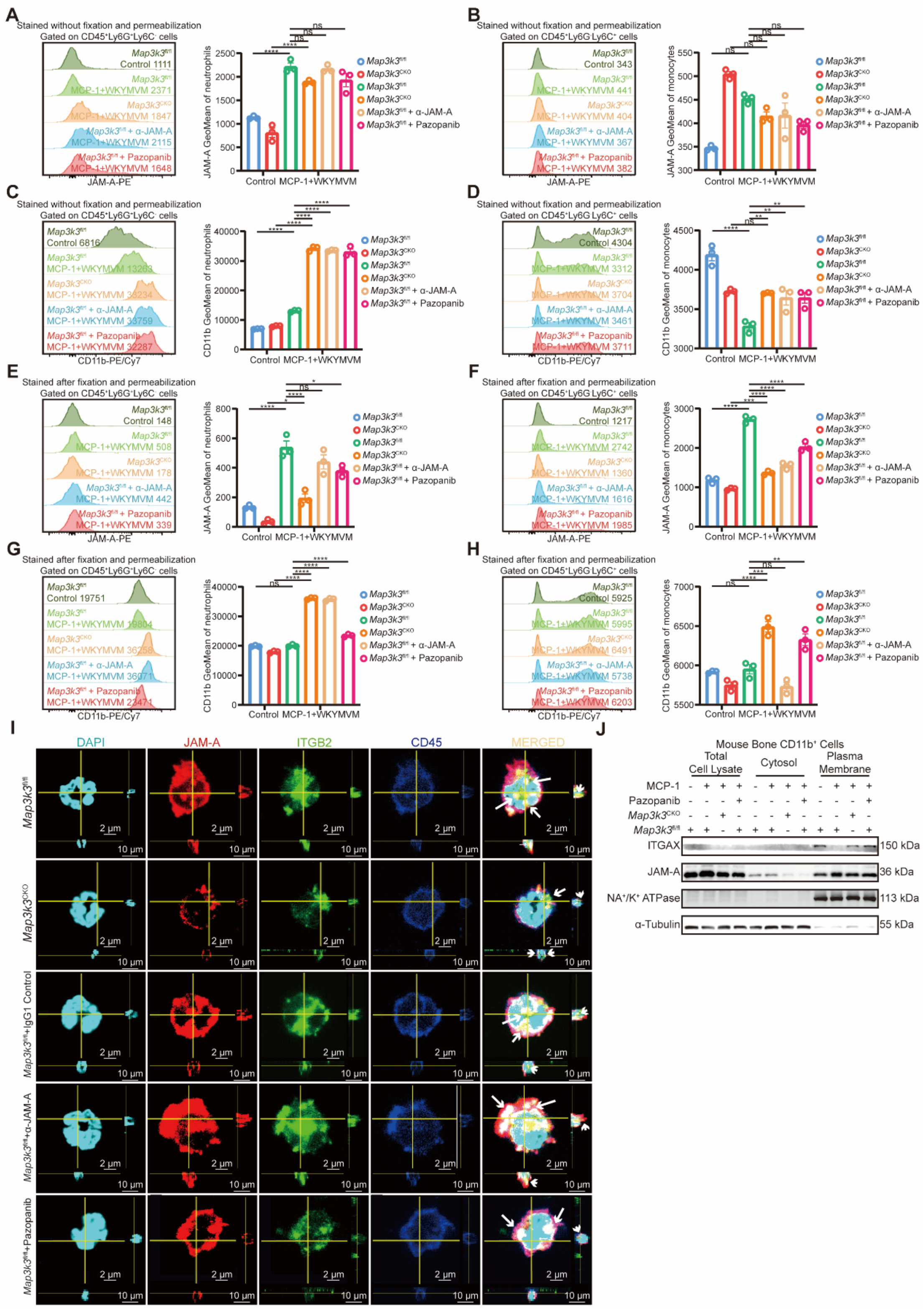

Figure S6. Reduced JAM-A led to dysfunction of integrin internalization

(A) Flowcytometric dot plot analysis of JAM-A expression in neutrophils without fixation and permeabilization (n=3 each, one-way ANOVA test with Tukey's multiple comparisons test). **(B)** Flowcytometric dot plot analysis of JAM-A expression in monocytes without fixation and permeabilization (n=3 each, Kruskal-Wallis's test with Dunn's multiple comparisons test). **(C)** Flowcytometric dot plot analysis of CD11b

(ITGAM) expression in neutrophils without fixation and permeabilization (*n*=3 each, one-way ANOVA test with Tukey's multiple comparisons test). **(D)** Flowcytometric dot plot analysis of CD11b (ITGAM) expression in monocytes without fixation and permeabilization (*n*=3 each, one-way ANOVA test with Tukey's multiple comparisons test). **(E)** Flowcytometric dot plot analysis of JAM-A expression in neutrophils after fixation and permeabilization (*n*=3 each, one-way ANOVA test with Tukey's multiple comparisons test). **(F)** Flowcytometric dot plot analysis of JAM-A expression in monocytes after fixation and permeabilization (*n*=3 each, one-way ANOVA test with Tukey's multiple comparisons test). **(G)** Flowcytometric dot plot analysis of CD11b (ITGAM) expression in neutrophils after fixation and permeabilization (*n*=3 each, one-way ANOVA test with Tukey's multiple comparisons test). **(H)** Flowcytometric dot plot analysis of CD11b (ITGAM) expression in monocytes after fixation and permeabilization (*n*=3 each, one-way ANOVA test with Tukey's multiple comparisons test). **(I)** Representative fluorescence images of integrin internalization by JAM-A stained for DNA (DAPI, cyan), JAM-A (green), integrin (ITGB2, red), and plasma membrane (CD45, blue) of cytokines stimulated bone marrow myeloid cells from *Map3k3*$^{fl/fl}$ mice or *Map3k3*^{CKO} mice, untreated or treated with IgG1, α-JAM-A, or Pazopanib. *3150, scale bar 2 μm; *630, scale bar 10 μm. Arrows point at JAM-A-integrin complex. **(J)** Western blots of ITGAX and JAM-A proteins in total cell lysate, cytosol, and plasma membrane of bone marrow myeloid cells from *Map3k3*$^{fl/fl}$ mice or *Map3k3*^{CKO} mice, untreated or treated with MCP-1 or Pazopanib. All data were displayed as mean ± SEM. ns, $P > 0.05$; * $P < 0.05$; ** $P < 0.01$; *** $P < 0.001$; **** $P < 0.0001$.

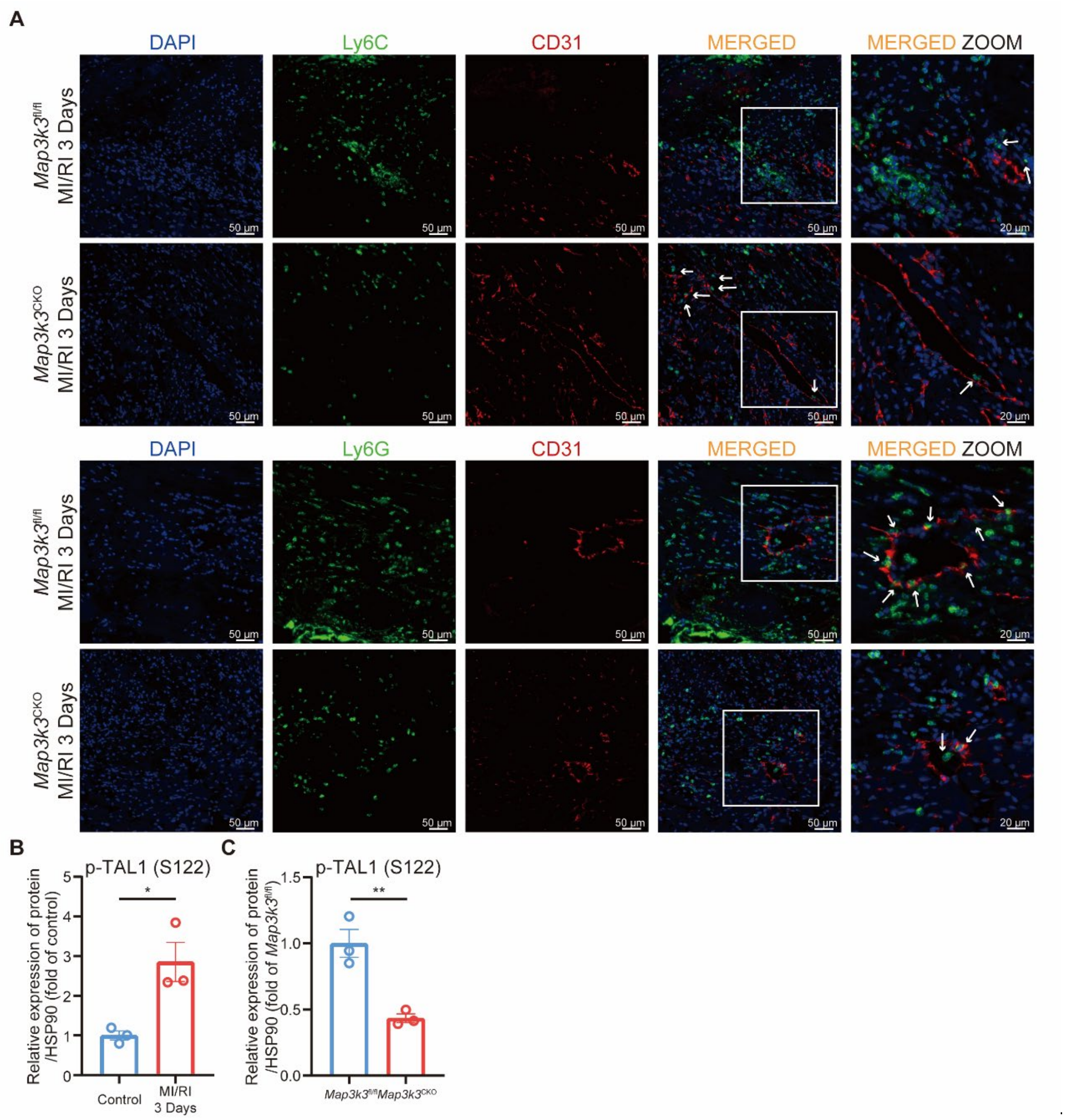

Figure S7. *Map3k3* deficiency increased adhesion and decreased diapedesis of monocytes and neutrophils from blood vessels to injured heart through p-TAL1 (S122)

(A) Representative fluorescence images of myeloid cells adhesion and diapedesis through blood vessels in MI/RI tissue sections stained for DAPI (blue), Ly6G/Ly6C (green), CD31 (red), *200, scale bar 50 μm; *400, scale bar 20 μm. Arrows pointed at adhered or migrating myeloid cells. **(B)** Relative protein levels of p-TAL1 (S122) of mouse blood myeloid cells from MI/RI mice and control mice (*n*=3, Mann-Whitney test, related to Figure 6K). **(C)** Relative protein levels of p-TAL1 (S122) of mouse blood myeloid cells from *Map3k3*$^{fl/fl}$ mice and *Map3k3*^{CKO} mice underwent MI/RI (*n*=3, unpaired *t* test, related to Figure 6L). All data were displayed as mean ± SEM. * $P < 0.05$; ** $P < 0.01$.

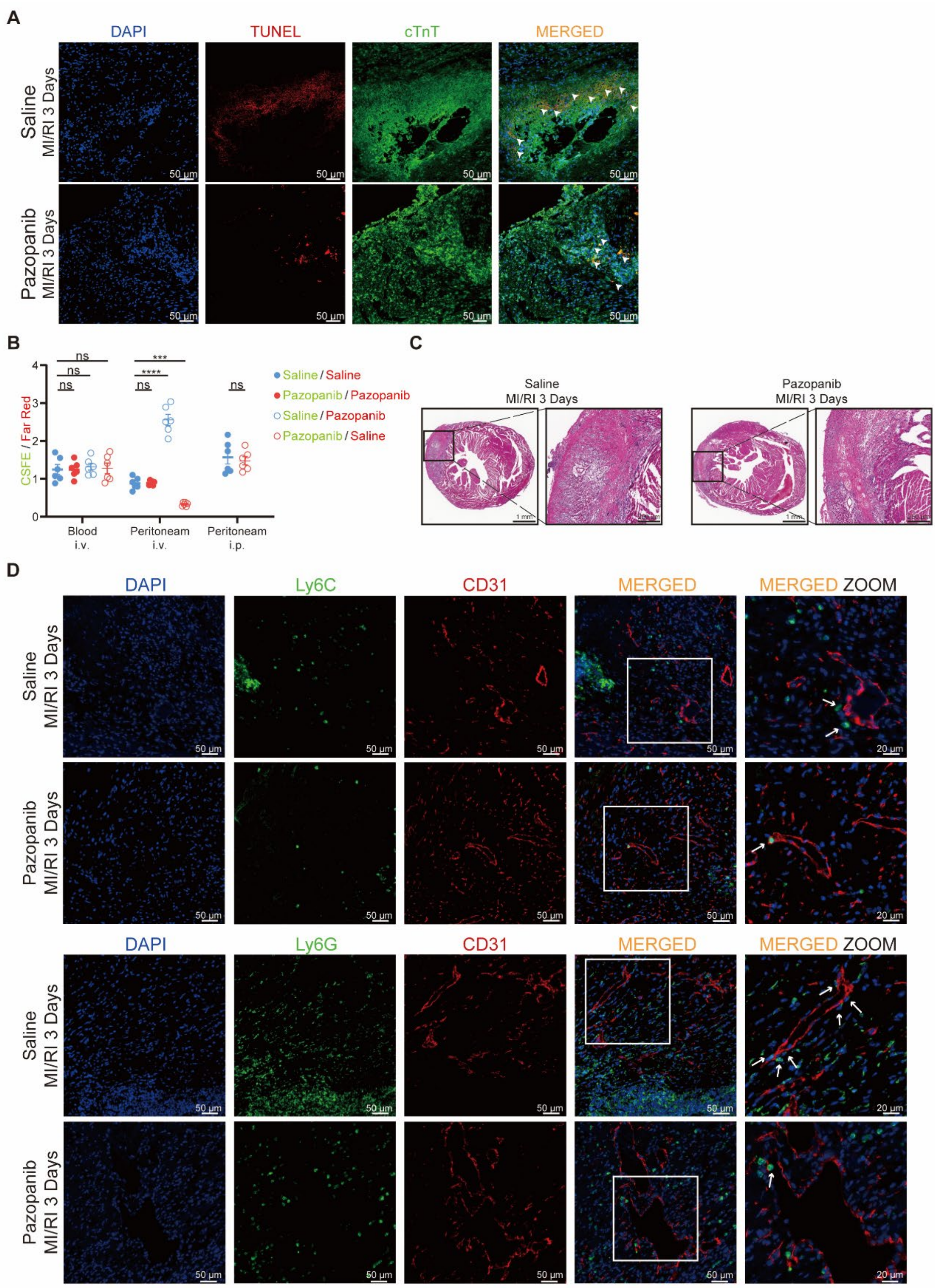

Figure S8. Pazopanib ameliorated MI/RI by increasing adhesion and decreasing diapedesis of monocytes and neutrophils

(A) Representative fluorescence images of apoptotic cardiomyocyte in MI/RI tissue sections stained for DAPI (blue), TUNEL (red), cTnT (green), *200, scale bar 50 μm. Arrows pointed at apoptotic cells (co-stained area of DAPI and TUNEL). **(B)** The ratios between CFSE and far-red-labeled cells collected from blood after i.v. (*n*=6 each, one-way ANOVA test with Tukey's multiple comparisons test), from peritoneum after i.v.

(*n*=6 each, Brown-Forsythe test with Dunnett's T3 multiple comparisons test), and from peritoneum after i.p. (*n*=6 each, unpaired *t* test). **(C)** Representative HE images of MI/RI tissue sections, *10, scale bar 1 mm; *40, scale bar 250 μm. **(D)** Representative fluorescence images of myeloid cells adhesion and diapedesis through blood vessels in MI/RI tissue sections stained for DAPI (blue), Ly6G/Ly6C (green), CD31 (red), *200, scale bar 50 μm; *400, scale bar 20 μm. Arrows pointed at adhered or migrating myeloid cells. All data were displayed as mean ± SEM. ns, $P > 0.05$; *** $P < 0.001$; **** $P < 0.0001$.

Supplementary Tables

Table S1. GPS6.0 prediction of TAL1 phosphorylation sites by MAP3K3

Substrate	Site	Kinase	Score	Cutoff
Mouse Tal1				
	S172	STE/STE7/MEK3/MAP2K3	0.0116	0.0105
	S12	CMGC/MAPK/ERK3/Erk3	0.0152	0.0146
	S172	STE/STE7/MEK3/MAP2K6	0.0161	0.0125
	S314	CMGC/MAPK/ERK3/Erk4	0.0268	0.0252
	T307	CMGC/MAPK/ERK3/Erk4	0.0274	0.0252
	S298	CMGC/MAPK/ERK3/Erk4	0.0275	0.0252
	S12	CMGC/MAPK/ERK3/Erk4	0.0289	0.0252
	S172	CMGC/MAPK/ERK3/Erk3	0.0298	0.0146
	S172	CMGC/MAPK/ERK3/Erk4	0.03	0.0252
	S314	STE/STE7/MEK1/MAP2K2	0.0449	0.0409
	S172	STE/STE7/MEK4/MAP2K4	0.0583	0.0432
	T307	STE/STE7/MEK7/MAP2K7	0.0586	0.0569
	T307	STE/STE7/MEK1/MAP2K2	0.0608	0.0409
	S172	STE/STE7/MEK7/MAP2K7	0.0639	0.0569
	S172	STE/STE7/MEK1/MAP2K2	0.065	0.0409
	S12	STE/STE7/MEK1/MAP2K2	0.0812	0.0409
	S172	STE/STE11/MEKK2/MAP3K3	0.1488	0.1362
	S7	CMGC/MAPK/ERK7/Erk7	0.5153	0.3792
	S12	CMGC/MAPK/ERK7/Erk7	0.5816	0.3792
	S172	CMGC/MAPK/ERK7/Erk7	0.5883	0.3792
	S122	STE/STE7/MEK1/MAP2K1	0.6642	0.233
	S122	CMGC/MAPK/ERK1/Erk1	0.9942	0.8675
Human TAL1				
	S172	STE/STE7/MEK3/MAP2K3	0.0111	0.0105
	S172	STE/STE7/MEK3/MAP2K6	0.0156	0.0125
	S12	CMGC/MAPK/ERK3/Erk4	0.0257	0.0252
	S172	CMGC/MAPK/ERK3/Erk3	0.0266	0.0146
	S300	CMGC/MAPK/ERK3/Erk4	0.0276	0.0252
	S172	CMGC/MAPK/ERK3/Erk4	0.0296	0.0252
	S300	STE/STE7/MEK1/MAP2K2	0.0414	0.0409
	S172	STE/STE7/MEK4/MAP2K4	0.0547	0.0432
	S172	STE/STE7/MEK1/MAP2K2	0.058	0.0409
	S12	STE/STE7/MEK1/MAP2K2	0.0695	0.0409
	S7	CMGC/MAPK/ERK7/Erk7	0.4368	0.3792
	S172	CMGC/MAPK/ERK7/Erk7	0.5415	0.3792
	S12	CMGC/MAPK/ERK7/Erk7	0.5827	0.3792
	S122	STE/STE7/MEK1/MAP2K1	0.7797	0.233
	S122	CMGC/MAPK/ERK1/Erk1	0.9939	0.8675

Table S2. Reagents and antibodies used in this study

Reagent name	Brand	Catalog Number	Dilution (if applicable)
EasySep™ Direct Human Neutrophil Isolation Kit	Stemcell	19666	
EasySep™ Direct Human Monocyte Isolation Kit	Stemcell	19669	
Trizol reagent	Invitrogen		
Reverse transcription kit	TaKaRa Bio		
qPCR SYBR Green Master Mix	Yeasen		
Pazopanib	Selleck	S3012	
EasySep™ Mouse CD11b Positive Selection Kit II	Stemcell	18970	
Type II collagenase	Worthington	LS004202	1.5 mg/mL
Elastase	Worthington	LS002274	0.25 mg/mL
DNase I	Worthington	LS002145	0.5 mg/mL
Red Blood Cell Lysis Buffer	Stemcell	20110	
Rat anti-mouse CD16/CD32 antibody	BD Pharmingen	553141	1:50 for FC
FVS510 dye	Biolegend	423102	1:1000 for FC
APC/cy7-conjugated anti-CD45	BD Pharmingen	557659	1:100 for FC<br>1:250 for IF
FITC-conjugated anti-Ly6G	Biolegend	127606	1:100 for FC
FITC-Rat IgG2α, κ antibody	Biolegend	400505	1:100 for FC
PE/cy7-conjugated anti-CD11b	BD Pharmingen	552850	1:100 for FC
BV421-conjugated anti-Ly6C	Biolegend	128032	1:100 for FC
BV421-Rat IgG2c, κ antibody	Biolegend	400725	1:100 for FC
Cytofix/Cytoperm Soln Kit	BD Pharmingen	554714	
MAP3K3 antibody	Abclonal	A3695	1:100 for FC<br>1:1000 for IF<br>1:1000 for WB
PE anti-rabbit antibody	Abcam	ab7007	1:1000 for FC
PE-conjugated anti-F11R	Biolegend	107803	1:100 for FC<br>1:250 for IF
DAPI	Beyotime	C1005	
Ly6G antibody	Servicebio	GB11229	1:1000 for IF
Ly6C antibody	Abcam	314120	1:1000 for IF
CD31 antibody	Abcam	ab182981	1:1000 for IF
cTnT antibody	Abclonal	ab8295	1:1000 for IF
In Situ Cell Death Detection Kit-Fluorescein	Roche	11684795 910	
TYR-570 Plus fluorescent dye	Recordbio	RC002	

TYR-520 Plus fluorescent dye	Recordbio	RC001	
MCP-1	Abclonal	RP01626	
WKYMVM	MCE	HY-P1120	
CFSE	Thermo Fisher	C34570	
Far-Red DDAO SE	Thermo Fisher	C34572	
Thioglycolate	Sigma	367-51-1	
Dual-Luciferase Reporter Assay Kits	PROMEGA	E1910	
TNF-α	Abclonal	RP01071	
6.5 mm Transwell® with 8.0 µm Pore Polycarbonate Membrane Insert	Corning	3422	
IgG1	MP Biomedical		
α-JAM-A	Santa Cruz	sc-53623	
CellTracker Green CMFDA	Yeason	40721ES50	
poly-L-lysine	Beyotime	C0313	
rabbit anti-ITGB2	Proteintech	10554-1-AP	1:2000 for IF
goat anti-rabbit IgG Alexa Fluor 488	Abcam	ab150077	1:500 for IF
Lipofectamine™ 3000	Thermo Fisher	L3000015	
RIPA lysis buffer	Beyotime	P0013C	
Immobilon western chemiluminescent HRP substrate	Millipore		
β-ACTIN antibody	KANGCHEN Biotech	KC-5A08	1:4000 for WB
HSP90 antibody	CST	4874	1:1000 for WB
BAX antibody	CST	2772	1:1000 for WB
CASPAS3-3 and Cleaved CASPASE-3 antibody	Proteintech	19677	1:1000 for WB
TAL1 antibody	Abclonal	A12927	1:1000 for WB
JAM-A antibody	Abclonal	A23909	1:1000 for WB
p-TAL1 (S122) antibody	Signalway Antibody	11814-1	1:1000 for WB
ITGAX antibody	Abclonal	A1508	1:1000 for WB
Na^+/ K^+ ATPase antibody	Invent Biotechnology	IN-SM005AB	1:1000 for WB
α-tubulin antibody	Abclonal	AC025	1:1000 for WB
Ubiquitin antibody	CST	14049	1:1000 for WB

p-S/T antibody	CST	9631	1:1000 for WB
HA tag antibody	Abcam	ab1424	1:1000 for WB
His-Tag antibody	CST	2365	1:1000 for WB
MG132	MCE	HY-13259	
Cycloheximide (CHX)	MCE	HY-12320	
SDS loading buffer	Beyotime	P0015	
Magnetic bead conjugated anti-MYC	ShareBio	SB-NM019	
Recombinant human MAP3K3 protein	Thermo Fisher	PV4194	
10* kinase buffer	CST	9802	
ATP	Beyotime	D7378	
